# Reverse GWAS: Using genetics to identify and model phenotypic subtypes

**DOI:** 10.1371/journal.pgen.1008009

**Published:** 2019-04-05

**Authors:** Andy Dahl, Na Cai, Arthur Ko, Markku Laakso, Päivi Pajukanta, Jonathan Flint, Noah Zaitlen

**Affiliations:** 1 Department of Medicine, UCSF, San Francisco, California, United States of America; 2 Wellcome Sanger Institute, Cambridge, United Kingdom; 3 European Bioinformatics Institute (EMBL-EBI), Cambridge, United Kingdom; 4 Department of Human Genetics, David Geffen School of Medicine, UCLA, Los Angeles, California, United States of America; 5 Institute of Clinical Medicine, Internal Medicine, University of Eastern Finland, Kuopio, Finland; 6 Kuopio University Hospital, Kuopio, Finland; 7 Center for Neurobehavioral Genetics, Semel Institute for Neuroscience and Human Behavior, UCLA, Los Angeles, California, United States of America; Stanford University School of Medicine, UNITED STATES

## Abstract

Recent and classical work has revealed biologically and medically significant subtypes in complex diseases and traits. However, relevant subtypes are often unknown, unmeasured, or actively debated, making automated statistical approaches to subtype definition valuable. We propose reverse GWAS (RGWAS) to identify and validate subtypes using genetics and multiple traits: while GWAS seeks the genetic basis of a given trait, RGWAS seeks to define trait subtypes with distinct genetic bases. Unlike existing approaches relying on off-the-shelf clustering methods, RGWAS uses a novel decomposition, MFMR, to model covariates, binary traits, and population structure. We use extensive simulations to show that modelling these features can be crucial for power and calibration. We validate RGWAS in practice by recovering a recently discovered stress subtype in major depression. We then show the utility of RGWAS by identifying three novel subtypes of metabolic traits. We biologically validate these metabolic subtypes with SNP-level tests and a novel polygenic test: the former recover known metabolic GxE SNPs; the latter suggests subtypes may explain substantial missing heritability. Crucially, statins, which are widely prescribed and theorized to increase diabetes risk, have opposing effects on blood glucose across metabolic subtypes, suggesting the subtypes have potential translational value.

## Author summary

Complex diseases depend on interactions between many known and unknown genetic and environmental factors. However, most studies aggregate these strata and test for associations on average across samples, though biological factors and medical interventions can have dramatically different effects on different people. Further, more-sophisticated models are often infeasible because relevant sources of heterogeneity are not generally known *a priori*. We introduce Reverse GWAS to simultaneously split samples into homogeneous subtypes and to learn differences in genetic or treatment effects between subtypes. Unlike existing approaches to computational subtype identification from high-dimensional trait data, RGWAS accounts for covariates, binary disease traits and, especially, population structure–important features of real genetic datasets. We validate RGWAS by recovering known genetic subtypes of major depression. We demonstrate RGWAS can uncover useful novel subtypes in a metabolic dataset, finding three novel subtypes with both SNP- and polygenic-level heterogeneity. Importantly, we show that RGWAS can uncover subtypes with differential treatment response: we show that statin, a common drug and potential type 2 diabetes risk factor, may have opposing subtype-specific effects on blood glucose.

## Introduction

Distinguishing subtypes can be essential for treatment, prognosis, and learning basic disease biology. For example, breast cancer has subtypes distinguished by tumor hormone receptor status that have different genetic risk variants, population structure, comorbidities, treatment responses, and prognoses [1, 2]. Many other common diseases have known, biologically distinct subtypes [3–7], often involving distinct tissues or biological pathways, including two diseases we study: major depression (MD) [8] and type 2 diabetes (T2D) [9, 10]. Genetically distinct subtypes can arise from gene-environment interactions [11–13]; gene-gene interactions [14, 15]; or disease misclassification, which is well documented but usually ignored [16, 17].

In this work we describe a novel method to learn and validate genetic subtypes in a two-step approach that we call reverse GWAS (RGWAS). In the first step, RGWAS infers subtypes by clustering multiple traits with a finite mixture of regressions method we designed specifically for large, multi-trait GWAS datasets (MFMR). The core assumption of MFMR is that the subtypes differ in distribution for many traits, which creates a subtype structure that can be detected with computational algorithms. In the second step, RGWAS assesses the causal *biological* distinction between the inferred subtypes by testing for genetic effect heterogeneity across subtypes, both at the SNP- and polygenic-levels. We also test effect heterogeneity for non-genetic covariates, like medical interventions, to assess *pragmatic* distinctions between subtypes [18–21].

RGWAS offers several important advances in both the identification and testing steps over other recent computational approaches to uncover subtypes [22–31]. First, unlike previous methods, the RGWAS identification step corrects for population structure, handles binary traits, and scales to tens of thousands of samples. Second, previous approaches to the testing step were badly confounded and/or under-powered to detect genetic heterogeneity in complex traits, while RGWAS offers calibrated *p*-values, polygenic subtype validation tests, and covariate adjustment. These advances substantially reduce both type I and type II errors in computational subtyping.

We first evaluate RGWAS through extensive simulations over a wide range of parameter settings and generative models, including several scenarios that violate our assumed model. In comparison to previous methods, as well as several novel methods and obvious extensions, we find that RGWAS is substantially more powerful, more robust, and better calibrated. We then validate RGWAS in real data by recovering a recently discovered stress subtype in MD, as well as known subtype-specific SNP effects.

Finally, we apply RGWAS to a metabolic cohort where subtypes are unknown *a priori* and find strong statistical support for genetic effect heterogeneity across dozens of complex phenotypes: first, a subtype-aware mixed model substantially increases heritability, from 20.7% to 30.2% on average across traits; second, we identify dozens of subtype-specific SNP effects that could not be discovered in standard analyses, including three SNPs with previously identified metabolic interactions; third, subtype-aware GWAS increases the number of hits, from 60 to 70 across traits. Crucially, we find that statin, a widely prescribed drug that may increase diabetes risk [32–34], has opposing effects on blood glucose across metabolic subtypes, which suggests that learned subtypes may have significant translational value.

## Results

### Reverse GWAS is calibrated and powerful in simulations

We examine the relative behaviors of RGWAS and other recent methods for computational subtype identification through application to simulated datasets. We simulated from a range of generative models and parameter settings meant to reflect many of the complexities of real data (Methods, Supplementary Section 3). Our baseline includes noise that is correlated across traits; large main subtype effects; 27 quantitative and 3 binary traits; and 12 SNPs containing null, homogeneous, and heterogeneous SNPs. We simulate datasets both under a *K* = 2 model and a *K* = 1 model where no subtypes of any sort are present. For each parameter setting we aggregate results from roughly 300 simulated datasets. We explore several variations to this baseline later.

For each simulated dataset we first cluster individuals (RGWAS step 1) into a subtype vector *z*. We focus on our Multi-trait Finite Mixture of Regressions (MFMR) approach (Methods) and three other, conceptually distinct approaches to cluster individuals into subtypes (Methods). First, we use Gaussian Mixture Models (GMM) to represent covariate-unaware methods, e.g. *k*-means [28, 31] and TDA [22, 26, 27]. Second, we consider a novel approach based on Canonical Correlation Analysis (CCA) that defines the subtype vector *z* as the top phenotypic CC. Third, we use the true *z* to show the best-case scenario with perfect subtyping (Oracle).

First, we observed that MFMR outperformed non-oracle methods in recovering true cluster identities across a range of simulations settings (S1 Fig). While this does increase confidence in MFMR, clustering accuracy is not a directly useful metric because true clusters are not known in practice. Moreover, we are primarily concerned with the significance and interpretation of our inferred subtypes, rather than merely their existence, which requires calibrated tests for effect heterogeneity between identified clusters. Hence our primary evaluation focuses on the false- and true-positive rates (FPR and TPR) for the standard SNP-subtype (*z*) interaction test in (3), conditioning on main subtype effects (RGWAS step 2). A polygenic alternative for RGWAS step 2 is presented below (Methods, [35]). We report a true positive if the SNP was simulated with heterogeneous effects across subtypes, and a false positive if the SNP was simulated with null or homogeneous effects.

**Fig 1:**
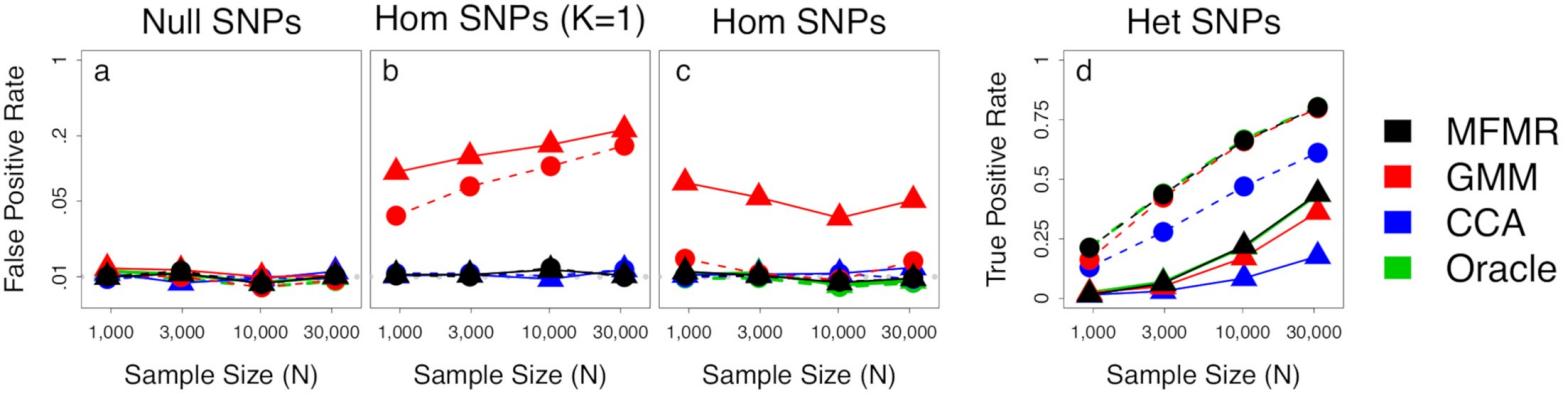
SNP heterogeneity tests at nominal *p* = .01. SNPs are either null (a); homogeneous (Hom, c); or truly heterogeneous (Het, d); we also test Hom SNPs in simulations with no subtypes (b). FPR is shown on the log scale. Hom SNPs explain 4% of variance and Het SNPs explain .4% (triangles) or vice versa (circles).

The results in Fig 1 show that interaction tests applied to MFMR subtypes are calibrated and almost perfectly obtain oracle power. Crucially, MFMR remains calibrated even when *K* = 1 (Fig 1b), so RGWAS discoveries reliably validate the existence of subtypes. Further, when *K* > 2 subtypes were simulated, MFMR with fixed *K* = 2 lost power but remained calibrated (S2a Fig). Together, this shows that MFMR is robust to misspecified *K*, though power increases when *K* is more accurate.

On the other hand, GMM is miscalibrated by an order of magnitude when *K* = 1, making it unreliable for interaction testing and, more broadly, validating the existence of subtypes (Fig 1b). One fundamental difference between MFMR and GMM is that only MFMR models covariates. Intuitively, ignored covariate effects confound true subtypes, hence GMM performs poorly when homogeneous effects are large or heterogeneous effects are small (Fig 1c, S2c–S2e Fig). Another difference between MFMR and GMM is that the former explicitly models binary traits, while the latter discards them and, concomitantly, loses power (S2b Fig, Methods).

Our novel application of CCA has low power but seems calibrated and, sometimes, seems to outperform even the oracle (e.g. S2c and S3 Figs). Unsurprisingly, this is a Pyrrhic victory: by smoothing over traits, CCA creates a bias that simultaneously increases FPR and TPR, which we show with theory (Supplementary Section 4) and simulation (S3 Fig). Moreover, the CCA subtypes poorly capture the true, discrete subtypes (S1 Fig). Broadly, CCA is often calibrated for testing the existence of heterogeneity, but it cannot determine which specific traits are truly heterogeneous and, moreover, has lower power than MFMR. We also tried using the top phenotypic PC for *z*, which performed like CCA except with lower power, and the top genetic PC [25], which had very low power (S4 Fig).

#### Simulations violating model assumptions

Having analyzed simulations from our assumed model, we turn to other generative models and parameter settings to assess the limitations of RGWAS. We first considered several direct variations to our baseline simulation. We drew *t*_5_-distributed noise, which has higher kurtosis than the Gaussian noise assumed by our model. We found that MFMR and CCA performed similarly as in the Gaussian case, but GMM became even more inflated (S5 Fig); nonetheless, we caution that MFMR, like linear regression, will not generally be calibrated under arbitrary noise distributions. Next, we assessed non-linear SNP effects and found that MFMR again remained roughly calibrated, but GMM and CCA were inflated for strong homogeneous effects; this held for two types of non-linear covariate transformations (S6 Fig), but we again caution that MFMR is not generally calibrated when covariates have non-linear effects. We also tried replacing the discrete subtypes *z* with a continuous *z* drawn from a Gaussian distribution. This exacerbated GMM inflation, but MFMR remained powerful and calibrated; however, when we doubled all heterogeneous effect sizes, MFMR became ≈ 4-fold inflated for large homogeneous covariates and *N* ≥ 30, 000 (S7 Fig), suggesting caution for MFMR in large datasets when large-effect, continuous subtypes may exist. Overall, modest amounts of these types of model misspecification cause relatively modest RGWAS bias, but stronger model violations will inevitably cause FPR inflation.

Second, we examined case ascertainment, which causes bias in many genetic testing settings [36–40]. We simulated “Case/Control” studies of a relatively rare disease, with population prevalence 20%, by ascertaining a 50/50 case/control dataset (S8 Fig). Little changed qualitatively, though all methods, even the oracle, had slight FPR inflation for large *N*. This modest level of inflation is expected because the ascertainment process violates the interaction regression model [41].

Third, we examined the robustness of MFMR to covariate/phenotype misclassification. Running MFMR requires specifying whether each available variable is a trait or a covariate (though SNPs are always covariates because of Mendelian randomization, and all the covariates in our simulation are SNPs). Intuitively, subtypes are clusters of covariate-adjusted traits. Variables that confound subtype structure, then, should included as covariates, while variables that may have different distributions between subtypes should be included as traits. Nonetheless, this distinction can be murky in practice, and so we perform simulations where we treat a covariate like a trait or vice versa. MFMR remained calibrated when swapping traits and covariates, unlike GMM, suggesting exact covariate/trait specification is not essential for valid inference with MFMR. However, MFMR did lose power, emphasizing the statistical utility derived from properly modelling the distinction between covariates and traits (S9 Fig).

Fourth, we studied the impact of SNP-subtype correlation (Supplementary Section 3.2), which is a known source of bias in genetic interaction tests [42]. We found that power held roughly constant for all methods as the G-E correlation increased from 0 to 1, but FPR did increase for all methods, including the oracle (S2f Fig). Nonetheless, the MFMR inflation is modest–always less than 3-fold–and MFMR was no more inflated than the oracle. Altogether, this simulation does recapitulate known biases from G-E correlation, but it does not suggest that RGWAS is more susceptible to these biases than standard tests for genetic interaction.

Finally, and most importantly, we examined the robustness of the clustering methods to population structure, which is routinely the primary confounder in genetic studies. We simulated a 50/50 mixture of two populations and 10,000 SNPs from a Balding-Nichols model with *F*_*ST*_ = .1 (Supplementary Section 3.3). We repeated our simulations using 12 randomly selected SNPs (out of the 10,000) and adding population main effects of varying strength. For MFMR and the oracle, we condition on three genetic PCs and their interactions with *z* in the step 2 tests. MFMR remains calibrated and powerful while CCA and GMM suffer substantial FPR inflation, even for completely null SNPs (S8 Fig). Using PCs in step 2 largely addresses the inflation from using GMM in step 1, but only when subtype structure is stronger than population structure; otherwise, the inferred GMM subtypes are non-trivially confounded by population structure. This is important because population structure is often stronger than subtype structure in reality, especially in multi-ethnic datasets, and because GMM produces false positives when subtypes are completely absent. Altogether, successful genetic subtype inference requires population structure adjustment both when inferring subtypes in step 1 and when testing their genetic significance in step 2.

### Positive control: A known major depression subtype

The results over the simulated datasets showed that RGWAS was powerful and calibrated across a wide range of parameter settings. To see if RGWAS could perform well in a real setting, we used CONVERGE [43], a major depression (MD) cohort with a recently discovered, genetically heterogeneous “Stress” subtype [44]. This serves as a positive control for genetic subtype discovery. In addition to having a known subtype, CONVERGE is ideally suited to RGWAS analysis because it recruited a large number (*N* = 9, 303) of deeply phenotyped (31 binary and 10 quantitative traits) Han Chinese women with recurrent MD and matched controls. Cases were carefully ascertained to minimize environmental heterogeneity, comparatively amplifying signals for biological heterogeneity. RGWAS analysis is also compelling because only a small number of genetic associations with MD have been found to date–consistent with the existence of genetically distinct disease subtypes–and there are few known genetic interactions in complex human traits.

**Fig 2:**
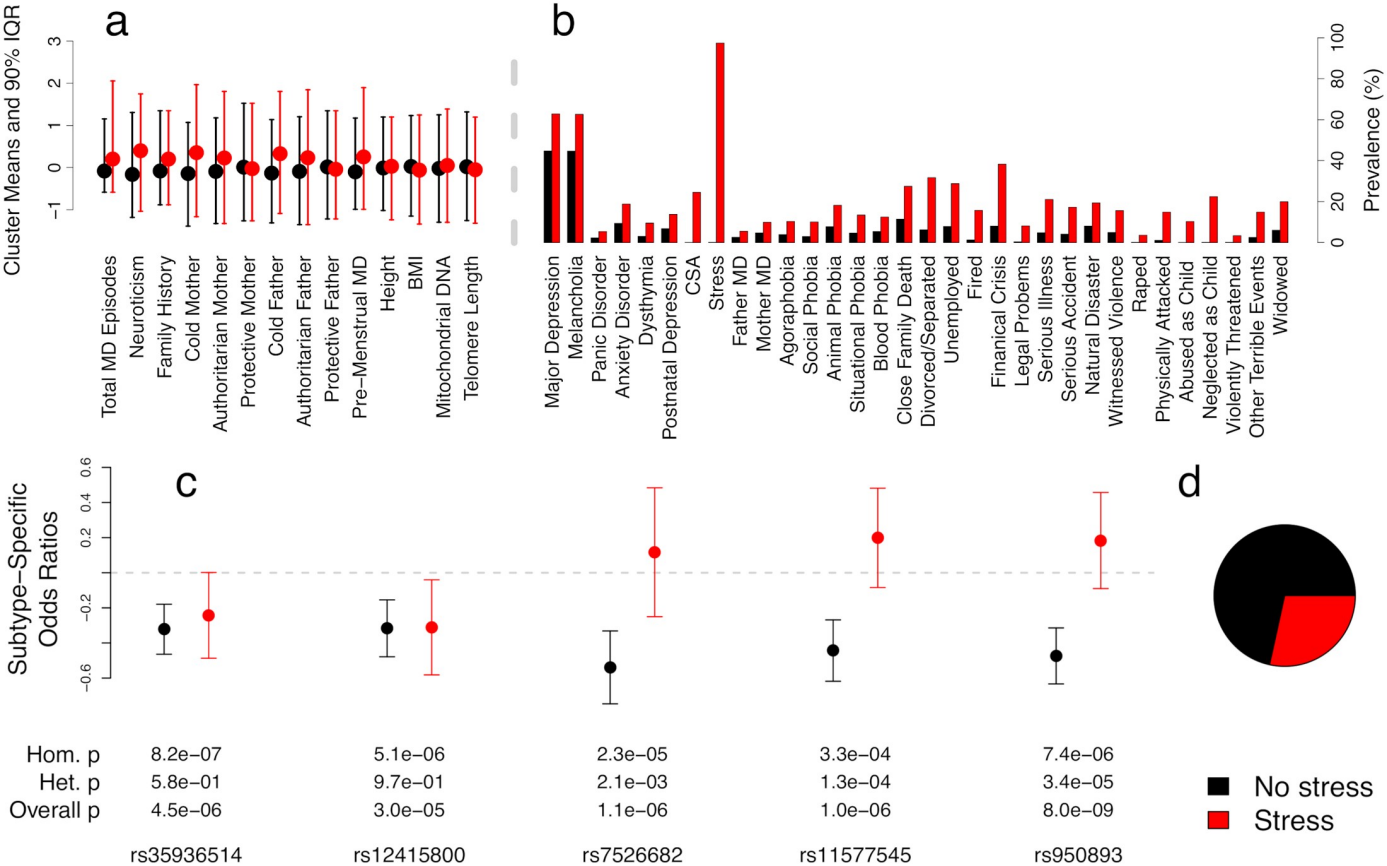
Genetic heterogeneity in the CONVERGE major depression dataset. Quantitative trait 90% inter-quantile ranges (a) and binary trait prevalences (b) are shown for each subtype. (c) Per-subtype odds ratios (±2 s.e.) for two SNPs discovered by (homogeneous) GWAS [43] (left) and three SNPs discovered using known subtypes [44] (right).

The inferred MFMR subtypes (RGWAS step 1) with *K* = 2 are summarized in Fig 2. We conditioned on age and ten genetic PCs as covariates, and we jointly imputed the covariates and traits (Methods). The MFMR method split the individuals into subtypes that distinguish the aggregate lifetime adversity measure “Stress”, which recovers the subtype chosen by domain experts [44]. While “Stress” is an obvious contributor to MD risk, there is no reason to expect MFMR would identify this as the key factor given we are studying a large number explicitly MD-relevant traits. Indeed, GMM did not split along this trait (squared correlation with “Stress” is .01), demonstrating “Stress” is not a trivially obvious subtype. The GMM clusters were not examined further as they have high FPR in simulations.

We tested five SNPs for effect heterogeneity (RGWAS step 2) across subtypes (Fig 2c) with our standard SNP-subtype interaction test. The first two SNPs (rs35936514 and rs12415800) were discovered in the initial GWAS [43] and we use as negative controls for heterogeneity. For positive controls, we use three SNPs (rs7526682, rs11577545, and rs950893) that we recently found to interact with “Stress” [44]. As expected, the homogeneous SNPs are nearly genome-wide significant, and RGWAS successfully determines which of the five SNPs are heterogeneous. Note that the heterogeneous SNPs show only modest homogeneous signal because they essentially have no effect in the “Stress” subtype. In previous work, we established that these subtypes have differential polygenic architecture using linear mixed models (*p* = .038); we also found suggestive polygenic score interaction and suggestive differences in the respective heritability estimates per subtype [44], further supporting the genetic distinction between “Stress” subtypes of MD.

We chose *K* = 2 using prior knowledge that MD can be split by (binary) “Stress”. We assessed this empirically by evaluating the MFMR likelihood on held-out data, which supported *K* > 1 subtypes (S10 Fig). *K* = 3 creates an MD-only subtype, so we do not pursue *K* ≥ 3.

### New metabolic subtypes with genetic and pragmatic significance

We next applied MFMR (RGWAS step 1) to metabolic traits measured in METSIM [45]. By combining genetic, environmental, metabolomic, and disease measurements, METSIM enables tracing the pathway from risk factors to metabolic consequences to altered disease risk. We studied 6,248 unrelated Finnish men. We used three binary traits: 854 samples had T2D, 3,526 had pre-diabetes (preT2D), and 541 had coronary heart disease (CHD); we excluded 15 samples with T1D. We used 13 broad quantitative traits and 228 nuclear magnetic resonance (NMR) metabolite measurements. We projected the NMR traits onto their top 6 PCs (capturing 77% of variance), which is a standard way to ease computation while retaining most of the information in the raw NMR traits. As covariates, we used three genetic PCs, age, age^2^, and smoking, alcohol, statin, diuretic, and beta-blocker use.

We study *K* = 3 to compromise between parsimony and the cross-validated log-likelihood, which broadly supports 1 < *K* ≤ 7 (S10 Fig). To test robustness to perturbations of the data, we used five-fold cross-validation. We found that 99.6% of originally co-clustered pairs (i.e. same likeliest subtype) remained together, showing that people from the same training population can be accurately assigned to existing subtypes.

**Fig 3:**
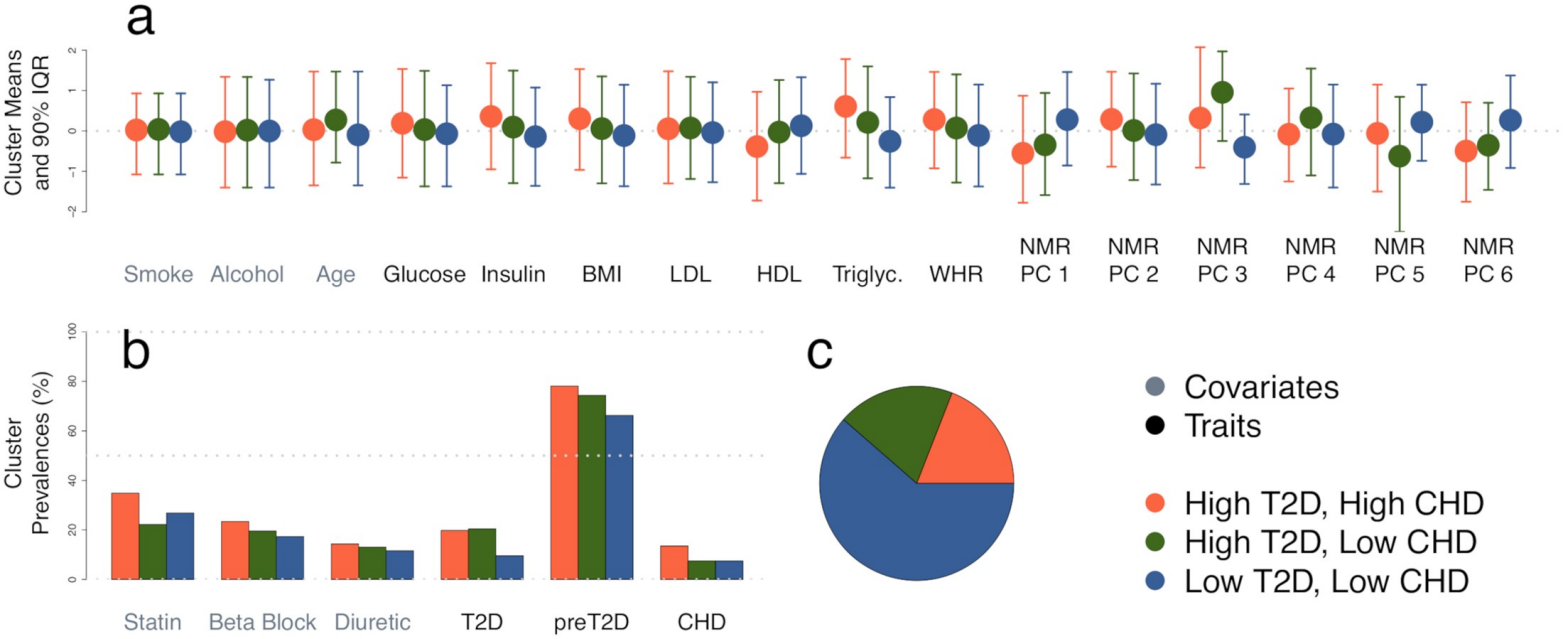
Three inferred metabolic subtypes in METSIM. (a) Quantitative and (b) binary distributions for covariates (grey labels, left) and traits (black labels, right). (c) Subtype sizes.

The three inferred metabolic subtypes are summarized in Fig 3. They primarily distinguish the metabolomic PCs, which are aggregates of 228 NMR traits. To elucidate the subtypes, we fit logistic regressions on the raw NMR traits, conditional on statin, and studied those with nominal *p* < .01 (despite [28, 30], these *p*-values are not calibrated). We first compared the large blue group to the combined orange and green groups, which suggested the blue group had less-esterified cholesterol in small HDL and higher histidine and relative amounts of omega-3 fatty acid. Next, comparing orange to green indicated orange had more free but less esterified overall cholesterol, especially in large LDL, and that orange has higher polyunsaturated fats and phenylalanine.

#### SNP level metabolic heterogeneity

**Fig 4:**
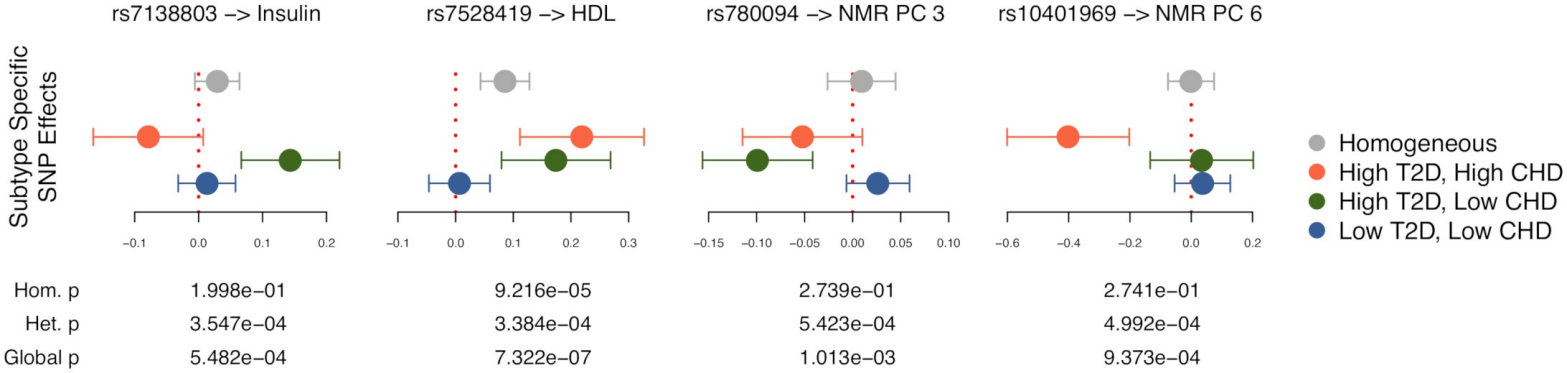
Metabolic subtype-specific SNP effects across the 16 traits used for subtyping. Subtype-specific effect estimates are shown ± 2 s.e. for significantly heterogeneous SNPs out of 81 known metabolic SNPs (*p* = .05/81).

We next sought to evaluate the subtypes for evidence of SNP effect heterogeneity on metabolic phenotypes (RGWAS step 2). However, with ∼6,000 samples, we do not have power for genome-wide heterogeneity tests. Instead, we test known metabolic GWAS SNPs–68 from T2D and 13 from CHD (Methods). We found four heterogeneous SNP-trait associations at *p* = .05/81 (Fig 4). The orange and green effect estimates had opposite sign for 2/4, and all blue estimates were near zero. This suggests that the blue group is a type of baseline and that partially overlapping biological pathways are specifically activated in the smaller groups.

These SNPs have several known metabolic interactions, providing additional evidence that the subtypes are meaningful. rs10401969 is a splice variant for *SUGP1* that affects downstream splicing in the gene targeted by statins, *HMGCR* [46]; it also interacts with an APOE SNP on fenofibrate response [47]. rs7138803 interacts with exercise for obesity [48] and features in an obesity score interacting with diet [49]. rs780094 interacts with another SNP for fasting glucose [50], suggestively interacts with diet [51], and is one of three SNPs in a risk score interacting with postprandial and post-fenofibrate cholesterol [52].

We additionally performed heterogeneity tests of these 81 SNPs directly over the 228 raw NMR phenotypes we used to construct the NMR PCs. We found 33 further SNP-trait interactions at *p* = .05/81 (S11 Fig). This included interactions between rs7138803 and 8 VLDL traits and insulin; rs7528419 and 16 VLDL traits and HDL; and rs780094 and triglyceride proportion in large VLDL. These results add confidence and interpretability to the interactions we discovered for NMR PCs 3 and 6. Further, we discovered 5 additional SNPs, not significantly heterogeneously associated with our 16 primary traits, including rs10885122 (interacting with medium HDL phospholipid percentage and HDL-3), rs1387153 (phospholid percentage in large and extra large VLDL), and rs6903956 (HDL).

To show an example of the utility of identifying subtypes, we performed a genome-wide interaction scan with the global, *K* df test [53]. This “GxE GWAS” test does not establish SNP heterogeneity, but it can increase power over ordinary GWAS when heterogeneity exists. GxE GWAS and GWAS give largely consistent results (Table 1, S12 Fig), as expected because the homogeneous and global tests are not independent. Nonetheless, GxE GWAS is a valuable complement to GWAS as it discovers 10 additional loci (though GxE GWAS misses 19/60 GWAS loci).

**Table 1:** Number of genome-wide significant loci for GWAS and GxE GWAS. Shared describes the number of loci that are significant in both the GWAS and GxE GWAS for the same trait (*r*^2^ < .2), while GWAS and GxE GWAS describe the loci unique to that association scan. No loci were found for CHD, insulin, or WHR. Both approaches found a single preT2D locus. We excluded NMR PC 5 because of GxE GWAS inflation.

	Gluc	BMI	LDL	HDL	TG	PC 1	PC 2	PC 3	PC 4	PC 6
GWAS	1	0	3	2	6	5	2	0	3	4
GxE GWAS	2	1	2	1	2	1	3	1	1	1
Shared	2	0	5	5	3	1	2	0	3	19

To mimic prior approaches, we performed a covariate-unaware GxE GWAS by using GMM subtypes (in step 1) and by excluding covariates from the heterogeneity tests (in step 2). Genome-wide tests were highly inflated for *K* ∈ {2, 3, 4}, usually obtaining effectively infinite λ_*GC*_ (S1 Table). Notably, λ_*GC*_ was even inflated for the binary traits, which were excluded from the clustering in step 1 in order for GMM to converge, emphasizing the subtlety and breadth of overfitting concerns in two-step testing. This inflation can easily be mistaken for strong, ubiquitous signal when evaluating only candidate SNPs, which is common in computational subtyping papers. The RGWAS λ_*GC*_ (using MFMR in step 1 and covariate-aware tests in step 2) were comparatively modest, with a maximum of 1.33 (after excluding NMR PC 5, with λ_*GC*_ = 1.83). Despite this modest inflation for some traits–which can be readily detected, and avoided, by evaluating λ_*GC*_–RGWAS results are usable, unlike the covariate-unaware results that mimic existing approaches. Similar conclusions hold for the global, *K* df test.

#### Polygenic metabolic heterogeneity

Identification of heterogeneous effects at individual SNPs provides both evidence of differential causal effects between subtypes and the specific loci that distinguish them. To complement these results, we additionally employed a polygenic linear mixed model (LMM) test for genetic subtype heterogeneity (RGWAS step 2). Such polygenic approaches have greater power to detect genetic signal than SNP-level tests, but they do not identify individual causal loci.

Briefly, the polygenic model estimates both the ordinary heritability and the subtype-specific heritability, the latter aggregating all subtype-specific SNP effects. For example, when subtypes are two sexes, the subtype-specific heritability aggregates all SNP effects that are active only in one sex (and, more generally, SNP effects that differ between sexes). For non-sex linked traits, the resulting sex-specific heritability is zero, hence nonzero subtype-specific heritability demonstrates the existence of subtype-specific genetic effects.

We fit the subtype specific LMM with IID GxEMM [35], which is a simple extension of a previous LMM for GxE [54] that accommodates probabilistic subtype membership. This is important because hard-assignment of individuals to subtypes discards information and fails to propagate step 1 subtyping uncertainty to step 2 subtype testing. We use the covariates used in our MFMR decomposition as fixed effects, as well as their interactions with subtype status [55]. We calculate variance explained after residualizing fixed effects [56]. We test for $h_{g}^{2}>0$ with a Wald test, and we test for $h_{het}^{2}=0$ with an LRT. For T2D, we exclude people with preT2D; for preT2D, we exclude people with T2D.

We report estimates for binary traits on the observed scale as there is no commonly accepted approach for rescaling heterogeneous heritability estimates to a liability scale. Similarly, we do not report p-values for these binary traits, to be maximally conservative. Nonetheless, we do observe that IID GxEMM increases the estimated heritability for preT2D and CHD, which is consistent with the existence of subtype-specific heritability.

**Fig 5:**
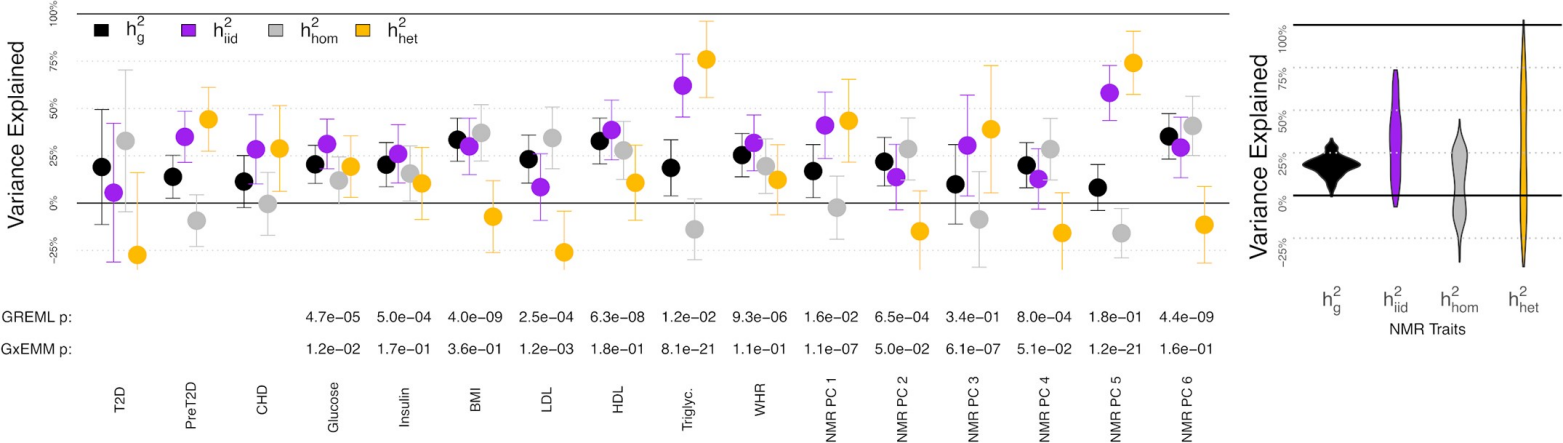
Polygenic heterogeneity in the inferred metabolic subtypes. Left: Point estimates ± 2 s.e. for traits used in MFMR clustering. Right: Across-trait estimate distribution for all 228 raw NMR traits. $h_{g}^{2}$ is the standard heritability estimate from GREML [54]. $h_{hom}^{2}$ and $h_{het}^{2}$ are the homogeneoues and subtype-specific heritability estimates from IID GxEMM, and $h_{iid}^{2}$ is the sum of $h_{hom}^{2}$ and $h_{het}^{2}$.

We found significant subtype-specific heritability for 4 of the 13 quantitative traits used in the MFMR decomposition (*p* < .01/13, Fig 5, left), giving strong evidence that our inferred subtypes tag meaningfully distinct biology. On average, these subtype-aware heritability estimates are 30.2%, compared to 20.7% for ordinary heritability estimates (S13 Fig). This shows that subtypes can mask substantial heritability across an array of traits. Intuitively, this increase in heritability derives from allowing subtype-specific effects that are ignored by homogeneous heritability: in the sex example, a SNP with exactly opposite effects in males and females has zero net contribution to homogeneous heritability, but it clearly contributes to the broader notion of subtype-specific heritability.

To expand to a larger set of traits and bolster confidence in widespread subtype-specific heritability, we repeated our IID GxEMM analysis on the 228 NMR traits (Fig 5, right). We found significant subtype-specific heritability for 104 of the 228 NMR traits (*p* < .01/228), dramatically increasing the number of significantly polygenically heterogeneous traits. On average, the subtype-aware heritability is 31.8% and the standard heritability is 17.5% (Fig 5), which are qualitatively comparable to the average heritabilities from the 13 quantitative MFMR traits.

#### Pragmatic metabolic heterogeneity

While identifying genetic heterogeneity is important for showing that subtypes have differential causal biology, identifying nongenetic sources of subtype heterogeneity can be pragmatically important. We tested for statin effect heterogeneity to assess the ability of the metabolic subtypes to differentiate medical intervention effects. Using our standard interaction test with *K* = 3 (Methods), only glucose had significant statin heterogeneity at *p* = 0.05/16 (*p* = 1.19 × 10^−4^). There is no obvious FPR inflation as statin has no other significantly heterogeneous effects across other traits (S2 Table). These results held after conditioning on T2D as a heterogeneous covariate in our step 2 test (statin-glucose interaction *p* = 2.95 × 10^−6^). Statin effect heterogeneity is further supported by tests with *K* = 4 (T2D-adjusted heterogeneity *p* = 9.67 × 10^−7^); for *K* = 2, however, the test is insignificant (*p* = .39), providing further evidence that *K* = 2 gives insufficient subtype resolution.

We next estimated the subtype-specific statin effects with our primary metabolic subtypes (derived treating statin as heterogeneous inside MFMR). We performed a heterogeneous linear regression on glucose conditional on our standard covariates and T2D, which indicated that statin increases blood sugar in most people–consistent with [32, 33]–but also that it may decrease glucose in the smaller, higher-risk orange and green groups.

Since METSIM measured two time points, we next tested the pragmatic ability of our baseline subtypes to predict conversion from preT2D to T2D. We fit logistic regression on time 2 T2D status for the 1,924 baseline prediabetics with time 2 data. Subtypes significantly predicted T2D conversion (*p* = 0.0036), with orange and green converting less often than blue, which remained after conditioning on our standard covariates (*p* = .031). This also demonstrates the subtypes persist at least partially over time, in contrast to prior, directly age-dependent T2D subtypes [57].

## Discussion

In a purely *descriptive* sense, inferring subtypes is easy: applying any clustering algorithm to any data produces subgroups. But existing methods cannot go beyond such descriptions because they are liable to downstream FPR inflation. By contrast, RGWAS is calibrated in simulation, recovers known MD subtypes, and produces *biologically* and *pragmatically* validated metabolic subtypes. RGWAS handles covariates, mixed binary and quantitative traits, and residual trait correlations.

There are several limitations to RGWAS. First, like other two step methods, RGWAS fails to propagate first-step uncertainty. Similarly, although we do not imagine there is a “true” *K*, more can always be done to better choose *K*. Also, while we have tested a variety of simple decompositions to learn subtypes, others may perform better, especially where domain-specific tools exist. In particular, MFMR is conceptually similar to a matrix factorization/depth-two linear network, suggesting inner layers of appropriate neural networks may define useful subtypes.

There are also specific limitations to our inferred stress subtypes in CONVERGE. First, our stress measurements were retrospective and self-reported, hence they may be biased by MD status. Second, our analysis was not entirely without domain supervision because we included the aggregate trait “Stress” that was previously manually constructed [44]. Nonetheless, RGWAS identified the key trait amongst dozens, unlike GMM, and our METSIM analysis demonstrates that RGWAS can be useful without any domain guidance.

The statin effect heterogeneity on glucose we found is consistent with previously reported interactions between statin and age [32] and genetically predicted LDL [34] on T2D, and also fenofibrate’s interaction with lipid levels on cardiovascular risk [58]. By contrast, large meta-analyses did not find inter-study statin heterogeneity [32, 33]. This suggests that statin heterogeneity largely exists within, rather than between, datasets. In the future it will be important to more precisely characterize the causal mechanism underlying statin heterogeneity. Although we expect such a mechanism to broadly replicate, the same is not necessarily true for our specific subtypes, especially as they rely on phenotypic PCs which are dataset-specific.

MFMR is only a first step toward genetic subtyping, and there are many possible extensions. Sparsifying penalties can be incorporated by replacing CM steps with calls to third-party software and could extend MFMR to higher-dimensional traits and covariates. A random-effect version of MFMR could improve power to detect polygenic subtypes, though computational issues are non-trivial. MFMR could also be adapted to count data, zero-inflation, higher-order arrays, or missing data. Theoretically, it would be interesting to let subtypes vary between traits, which MFMR can capture only non-parsimoniously by choosing large *K*. Instead of an i.i.d. prior on *z*, MFMR could model *z* with a multinomial logistic regression to estimate, test, and correct for effects on *z*, which can be directly interesting [59]; this can also be important for correcting bias from G-E correlation [42]. Or, instead, we could use a continuous prior on *z* with a factor analysis model [23]. (We note, however, that RGWAS copes reasonably well with modest G-E correlation and continuous *z* in our simulations.) Finally, MFMR could be applied only within diseased individuals to directly define subtypes of disease, though this requires fundamentally different step 2 tests (c.f. [60]).

Our polygenic approach to subtype validation with GxEMM provides a much needed power advantage over SNP-level heterogeneity tests at the cost of resolution; conceptually, polygenic risk score tests lie between [61, 62]. But SNP-level precision is not needed to meet our criterion for biologically meaningful subtypes, making GxEMM invaluable for subtype validation. Nonetheless, there are many limitations to our approach that we will address in future work. First, GxEMM can confuse non-linear effects for heterogeneity. Similar issues arise in generalized linear models, where the existence of effect heterogeneity depends on the choice of link function. Some forms of non-linearity can be accommodated [63], including ascertained case/control data where cases are over-sampled from the population [36–40]; we are working to extend these approaches to leverage subtype heterogeneity. Second, IID GxEMM assumes that each subtype has equal heritability and equal noise levels, which may not hold in practice for many meaningful subtypes. Several approaches exist to relax these assumptions to varying degrees [35, 64, 65], which reduce bias, provide richer characterizations of subtypes, and will be important to pursue in future work. However, this increased generality comes with higher estimation error for individual parameters, and we consider the results presented in our paper conservative alternatives to these richer models.

Although we focused on fixed- and random-effect interaction tests to establish heterogeneity between subtypes in RGWAS step 2, it may also be useful to apply recent, complementary heterogeneity tests. For example, Subtest could be used to assess differences between *K* = 2 disease-only subtypes [60]. For large *K*, on the other hand, StructLMM is a natural complement to GxEMM: the latter is more powerful because it uses genome-wide information and a richer GxE model, but the former has SNP-level resolution and scales to dramatically larger *N* and *K*. Similarly, large-*K* subtypes could be post-processed with hierarchical clustering and tested with TreeWAS [62]. Broadly, any heterogeneity test can be used in the second step.

In the future, we will actively encourage MFMR to prioritize tissue-specific subtypes by incorporating tissue-specific genetic risk scores as heterogeneous covariates. This is particularly interesting for traits, including metabolic diseases [15, 66], that have disparate genetic risk factors acting through distinct cell types, tissues, or biological processes. Subtypes that differentiate biological modes of action at this systems-level would be more easily interpretable and useful for basic research and precision treatment. In larger datasets, it may also be interesting to evaluate subtype-specific enrichments in heritability explained per tissue or cell type [67].

Finally, as MFMR seeks clusters that are unaffected by confounders like population structure, age, or sex, it may be useful for clustering in settings where protecting certain information is important for privacy or fairness [68]. In this sense, MFMR is to GMM roughly as AC-PCA [69] or contrastive PCA [70] are to ordinary PCA.

## Methods

### Ethics statement

CONVERGE: The study protocol was approved centrally by the Ethical Review Board of Oxford University (Oxford Tropical Research Ethics Committee) and the ethics committees of all participating hospitals in China. All participants provided written informed consent.

METSIM: The Ethics Committee of the University of Eastern Finland and Kuopio University Hospital approved the METSIM study, and this study was conducted in accordance with the Helsinki Declaration. All participants provided written informed consent.

### RGWAS step 1: Clustering with MFMR to find subtypes

Reverse GWAS first clusters samples into subtypes (step 1) and then tests for covariate effect heterogeneity between subtypes (step 2). RGWAS always uses MFMR in step 1. Step 2 always features interaction tests between subtype membership and focal covariates, which may be non-genetic, a SNP, or all SNPs in the genome.

We derive a novel clustering algorithm, multitrait finite mixture of regressions (MFMR), beginning from the standard regression model for interaction. Assuming a single quantitative trait *y*, covariates *X*, discrete subtypes *z*, and a focal covariate *g* putatively interacting with *z*, our model is:

$$\begin{array}{c} y_{i}=X_{i},\alpha +\gamma_{z_{i}}+g_{i}\beta_{z_{i}}+\epsilon_{i} \end{array}$$ (1)

*X*_*i*_, is a vector of *Q* control covariates, like genetic PCs or sex, with homogeneous effect sizes *α*. *z*_*i*_ ∈ {1, …, *K*} is a *K*-level factor specifying the subtype for individual *i*, and *γ*_*k*_ are the subtype main effects. *β* is the vector of subtype-specific *g* effect sizes. We say *g* is homogeneous if *β*_1_ = … = *β*_*K*_; otherwise, *g* is heterogeneous. We assume *ϵ* is i.i.d. Gaussian with mean zero.

Our full MFMR model generalizes (1) in several complementary directions. First, we learn the subtypes (*z*) rather than assume they are known (giving a Finite Mixture of Regressions, FMR) by assuming *z*_*i*_ are i.i.d. Categorical:

$$\begin{array}{c} P\left(z_{i}=k|p\right)=p_{k}\;\;\text{for}\;k=1,\ldots ,K \end{array}$$ (2)

Second, we generalize the single trait *y* to a matrix *Y* of Multiple traits (MFMR), which adds power for subtypes that affect the distribution of many traits. This power is important in practice because genetic interactions are often weak but phenotypic relationships are often strong. We also generalize the single heterogeneous covariate *g* to a matrix *G* of multiple covariates, which adds power when there are many heterogeneous effects.

Finally, we model binary traits with probit link functions to mitigate the spurious local modes that plague methods like *k*-means. For example, this issue led others to discard roughly half their data *post hoc* [31]. The full multi-trait probit model is computationally prohibitive even for modest *B*, which we address with a novel conditional independence assumption. This induces constraints in our optimization which we solve analytically with block matrix identities (Supplementary Section 2.3).

Computationally, we fit MFMR with an Expectation Conditional-Maximization (ECM) algorithm. Our ECM generalizes standard EM for Gaussian Mixture Models. Both iterate between *z* updates in E-steps and parameter updates (e.g. *α* and *β*) in (C)M steps.

When fitting MFMR in step 1, a covariate that will be tested for heterogeneity in step 2 can either be ignored (MFMRX), included in *X* (MFMR, our default), or included in *G* (MFMR+). In Gaussian mixture models, covariates can only be ignored (GMM) or added as traits (GMM+) [31]. MFMR+ and GMM+ overfit in simulations, inflating the FPR; conversely, MFMRX and GMM underfit homogeneous covariates, which also inflates FPR (S4 Fig). MFMR strikes a balance: the homogeneous effect is adjusted but subtypes are not tuned to the heterogeneous effect. This resembles a score test as the alternate is tested by evaluating only the null. Nonetheless, small-effect covariates, like SNPs, can be safely ignored within MFMR, enabling genome-wide testing with MFMRX [71]. For simplicity, we refer only to MFMR throughout our paper, but we use the MFMRX test for real human SNPs (in CONVERGE and METSIM, but not the simulations) as they likely have negligible effects; however, we use the MFMR test for statin in the main text because it has large metabolic effects.

We note that MFMR generalizes several well-known models. If binary traits and *X* are excluded and the covariates *G* are reduced to an intercept, MFMR becomes GMM. When *P* = 1 and *z* is known, MFMR becomes a standard gene-environment interaction (GxE) model with discrete environments/subtypes. Finally, if *P* = 1 and *β*_*k*_ = *β*_0_ for all *k*, MFMR reduces to linear/probit regression.

### RGWAS step 2: Calibrated tests to validate subtypes

For simplicity, we assume there are *K* = 2 subtypes and just one interacting covariate, *g*. These assumptions mean the output from step 1 is just a vector *z*, where *z*_*i*_ is the subtype 1 probability for sample *i*, and that the interaction model takes a simple form:

$$\begin{array}{c} y∼\tilde{X}\alpha +z\gamma +g\delta +(g*z)\beta +\epsilon \end{array}$$ (3)

$\tilde{X}$ collects all background covariates, like genetic PCs, unlike existing subtype validation tests that largely ignore population structure [24, 26, 29, 31]. * is element-wise multiplication, but it can easily be generalized to allow *K* > 2 and a matrix *G* instead of a single covariate *g*.

We consider three tests for *g*: the homogeneity test for *δ* ≠ 0 given *β* = 0; the heterogeneity test for *β* ≠ 0 with free *δ*; and the global test for *δ*, *β* ≠ 0 [53]. The homogeneity test has 1 degree of freedom (df), the heterogeneity test has *K* − 1 df, and the global test has *K* df. We focus on the heterogeneity test, which establishes that *g* has differential effects across subtypes and thus that the subtypes differ in causal biology (if *g* is genetic) or pragmatically (e.g. if *g* is a treatment). We also investigate the global test’s ability to increase power over the typical homogeneous test in the GxE GWAS analysis in Table 1. We assume *ϵ* is i.i.d and test with linear or logistic regression.

#### Polygenic step 2 tests

We also developed a polygenic version of the interaction test in (3) to jointly model and test *δ* and *β* across all SNPs with random effects. When there are no interaction effects, i.e. *β* = 0, this gives exactly the standard GREML approach to estimate heritability from genome-wide similarity across unrelated samples [56]. GREML accomplishes this by modelling each SNP’s homogeneous effect, *δ*, as a small Gaussian variable, and then aggregating the size of each SNP’s *δ* across the genome to estimate the total genetic contribution to phenotypic variability, i.e. heritability.

Polygenic interaction models go further by also giving the interaction effect, *β*, a random effect distribution. As the homogeneous mixed model used in GREML aggregates the *δ* estimates across the genome to estimate homogeneoues heritability, the interaction mixed model aggregates the *β* estimates along the genome to estimate the heterogeneous heritability explained by subtype-/environment-specific genetic effects. The latter provides an estimate of subtype-specific heritability, which can be combined with the homogeneous heritability estimates to partition broad-sense heritability into shared- and subtype-specific components. This approach was pioneered for unrelated humans in [54], but this model assumes that each sample is deterministically assigned to a single environment/subtype. Because our subtype assignments are probabilistic, we use GxEMM to fit subtype-specific heritabilities [35], which accommodates arbitrary environmental covariates.

Polygenic interaction tests are essential for genetic subtyping at modest sample sizes because the test for nonzero subtype-specific heritability is much more powerful than testing individual SNPs in complex traits. Polygenic interactions can demonstrate that subtypes have partially distinct genetic bases even when power is too low to discover individual subtype-specific SNP effects.

### Other approaches to infer subtypes

We develop a novel subtyping approach by applying CCA to *G* and the joint binary and quantitative phenotype matrix (*Y*^*b*^: *Y*), each column-wise centered and scaled, and taking *z* to be the top phenotypic CC. CCA (and phenotypic PCA) defines *z* as a linear trait combination. We prove that this causes the interaction tests to have inflated FPR when a mixture of heterogeneous and homogeneous traits are studied (Supplementary Section 4), which is likely in practice. Nonetheless, sparse estimators can resolve this problem in some theoretical settings, and CCA is computationally efficient (S1 Fig).

We also tested GMM, which models samples as draws from one of *K* multivariate Gaussians. We fit GMM to the quantitative traits with a standard EM algorithm [72]; in early tests where binary traits were included, GMM often failed to converge, or converged to exactly coincide with one of the binary traits, even with multiple random restarts. We consider GMM similar, in the sense of covariate-unawareness, to *k*-means, which struggles even more with binary traits, and TDA, a proprietary package.

Most similar to MFMR, LIMMI aims to identify GxE with unknown E in gene expression [23]. Beyond many technical differences, LIMMI and MFMR are built for disjoint scenarios: MFMR only fits tens of traits, but LIMMI only fits hundreds of samples, preventing its use in our setting.

### METSIM dataset

We selected metabolically relevant SNPs by taking published GWAS SNPs for T2D or CHD. We used the 153 T2D SNPs in Table 1 of [73] as known T2D SNPs. We had genotyped 86 of these SNPs, which we reduced further to 68 roughly independent SNPs (*r*^2^ < .1). We used the 65 CHD SNPs in S2 Table of [74] as known CHD SNPs, 13 of which we genotyped (all *r*^2^ < .1). We filtered the original 10,070 person dataset so all pairwise kinships were below 0.05, as in [56].

### Phenotype imputation

We imputed missing data before running MFMR in CONVERGE. We jointly imputed covariates and traits with a sample-wise i.i.d. Gaussian model (MVN-impute from [75]). We thresholded imputed entries in *Y*^*b*^ to {0, 1} in order to retain the downstream logistic regression framework. By contrast, discarding samples with any missing data reduces sample size by roughly half and the known positive SNP interactions were no longer recovered.

We imputed METSIM similarly, including all 228 NMR traits at the imputation step. We used softImpute to accommodate the wide matrix [76].

We note that complete-data analyses performed in similar contexts substantially reduce sample size, e.g. [31] discard 39% of their samples.

### Code availability

RGWAS is implemented in the simple, free rgwas R package, available with a vignette at https://github.com/andywdahl/rgwas.

All summary data and code necessary to reproduce the main and supplementary figures and tables are available at: https://github.com/andywdahl/rgwas-scripts.

## Supporting information

S1 Text Supplementary note. Full description of MFMR model and EM algorithm. Also includes full descriptions of simulations and proofs about PCA/CCA subtype estimators.
(PDF)

S1 Fig Running time and subtype estimation accuracy in simulations. Left: Average running times in main Fig 1 (excluding failed GMM runs). Right: Clustering accuracy for simulations without (‘Quantitative’, as in main Fig 1) and with ascertainment (‘Case/Control’, as in S2 Fig). We measure accuracy with adjusted Rand index, which varies from 0 (random guessing) to 1 (exact match). We compute the index only across pairs from a random 300 subsamples, reducing computation roughly ≈ 10^5^-fold when *N* = 100, 000. Accuracies are estimated for roughly 300 simulations per point in the plot. MFMR+ is shown for simplicity because MFMR gives different clusters per tested SNP.
(TIF)

S2 FigSimulations varying several further parameters.Tests for truly heterogeneous SNPs are shown in the top 6 panels (a-f), and the corresponding tests for SNPs with only homogeneoues effects are shown in the below 6 panels (g-l). *K* is the number of true, simulated subtypes and *B* is the number of binary traits. *ρ*_*GE*_ is the gene-subtype correlation term, with *ρ*_*GE*_ = 0 giving non-heritable subtype statuses and *ρ*_*GE*_ = 1 giving perfectly heritable subtypes. $h_{hom}^{2}$, $h_{het}^{2}$, and $h_{z}^{2}$ are the variances explained by homogeneous SNPs, heterogeneous SNPs, and main subtype effects, respectively. As in main Fig 1, solid lines have $\left(h_{hom}^{2},h_{het}^{2}\right)=\left(4\%,.4\%\right)$, and dashed lines are reversed; in (d,e), line types define only the *h*^2^ term not governed by the x-axis. In (a), all methods fit *K* = 2 subtypes; there is no true heterogeneity for *K* = 1, where the oracle is not defined, and for *K* > 1 and the oracle picks a true cluster at random. Generally, increasing the heterogeneous factors ($h_{het}^{2}$ and $h_{z}^{2}$) makes subtyping easier, while increasing $h_{hom}^{2}$ makes subtyping harder.(TIF)

S3 Fig Simulations where SNP heterogeneity only exists for some traits, for which they are only homogeneous. Left: the tested trait has no genetic heterogeneity or main subtype effect. Center: the tested trait has only a main subtype effect but no heterogeneity. Right: the full heterogeneity simulation. Linear subtype estimators (CCA and *Y* PC) are not trait-specific.
(TIF)

S4 Fig Main Fig 1 with further subtyping methods. MFMR+ varies MFMR by treating the tested SNP as heterogeneous. GMM+ varies GMM by including the SNPs as traits when clustering. As expected, MFMR+ and GMM+ are miscalibrated. GMM+ often fails to converge, especially for *N* ≥ 10, 000 (we evaluate only the converged runs). The other methods, with low power, define subtypes as the top PC of *Y* or *G*, optionally thresholded to be binary (“G PC+disc”).
(TIF)

S5 Fig Simulations with non-Gaussian noise. Purely homogeneoues simulations, without subtypes, where the noise, *ϵ*, has marginal *t*_5_ distributions. *ϵ* is simulated by drawing i.i.d. *t*_5_-distributed random variables, arranging into an *N* × *P* matrix, and then right-multiplying with Σ^1/2^, where Σ is the noise covariance matrix and is drawn as in the main simulations in main Fig 1.
(TIF)

S6 Fig Simulations with non-linear homogeneous effects. Purely homogeneoues simulations, without subtypes, where SNPs truly have a non-linear effect. In (a-d), the SNPs are squared before use in MFMR, so that the true SNP and the utilized covariate (i.e. SNP^2^) have zero correlation. In (e-h), the true SNPs are exponentiated before inclusion in MFMR, so the true SNP effect is log-linear. Results are partitioned by whether the tested traits are quantitative or binary, as well as by whether the true SNP effect is null or homogeneous.
(TIF)

S7 Fig Simulations with continuously-varying subtypes. *z* is chosen to be Gaussian. Top: Effect sizes are chosen so that power roughly matches main Fig 1; it is not trivial to directly convert effect sizes from the discrete *z* simulations. Bottom: All heterogeneoues effect sizes are doubled relative to top panels.
(TIF)

S8 Fig Alternate versions of Fig 1. Top: SNP effect heterogeneity tests are applied to binary traits, not quantitative traits as in main Fig 1. Even though GMM only clusters the quantitative traits, tests for the (correlated) binary traits are miscalibrated. Middle: a 20% population prevalence binary trait is ascertained to have 50% in-sample prevalence and then tested. Bottom: population structure is added and MFMR, Oracle and GMM-PC test conditional on three genetic PCs; GMM and GMM-PC use the same subtype estimator.
(TIF)

S9 Fig Simulations where some covariates and traits are swapped. Simulation modification where decompositions falsely treat a trait as a SNP/covariate (a,b,e,f,i) or vice versa (c,d,g,h,j). (a-d) No genetic or main subtype heterogeneity is simulated, so that the positive heterogeneity associations are unambiguously false. We test both the variable that we misplace (a,c) and the correctly place SNP/covariates and traits (b,d). (e-j) Simulations are drawn as in main text Fig 1, with *K* = 2. (e-h) Tests are shown for the misplaced trait/covariate in (e,g); for the truly homogeneous SNPs in (f,h); and for the truly heterogeneous SNPs in (i,j).
(TIF)

S10 Fig Out-of-sample likelihood varying *K* in CONVERGE (left) and METSIM (right). Samples are split into 5 folds; parameters are fit holding one fold out; the parameters’s likelihood is evaluated on the held out fold; and the process is repeated for each fold. The log-likelihoods are shown relative to the baseline likelihood of each fold at *K* = 1; this is analogous to using likelihood ratio statistics to compare a general *K* to the null with *K* = 1. The average across folds are shown in red, and the maximizer of *K* is highlighted in green.
(TIF)

S11 Fig Metabolic subtype-specific SNP effects across all 228 NMR traits. SNP-phenotype pairs where the test for effect heterogeneity across subtypes is significant at *p* = .05/81. We test all 228 NMR-based metabolomic traits here rather than using their top PCs as in main Fig 4 and the MFMR decomposition used to learn subtypes. Per-subtype estimates and standard errors are provided in colors as in main Fig 4.
(TIF)

S12 Fig Comparison of the −log_10_(*p*)-values for ordinary GWAS (x-axis) and our novel GxE GWAS (y-axis). Guide lines are drawn at *p* = 5 × 10^−8^, the conventional GWAS threshold. Each point is a SNP, and colors indicate which analyses were significant for the SNP. T2D, CHD, WHR and insulin are omitted because they have no genome-wide significant hits in either analysis; preT2D is omitted because the only hit is shared between both analyses. NMR PC 5 is omitted because it is badly inflated in GxE GWAS (λ_*GC*_ = 1.83); this trait has one hit in GWAS.
(TIF)

S13 Fig Comparison of GREML and IID metabolic heritability estimates. Left: total IID GxEMM heritability (which adds the homogeneous and heterogeneous estimates) compared to the ordinary heritability estimated with GREML. Right: histogram of per-trait heritability increases from replacing GREML with IID GxEMM.
(TIF)

S1 Table λ_*GC*_ for GWAS, MFMR GxE GWAS, and GMM GxE GWAS. GWAS means the standard regression approach conditioning on known covariates and genetic PCs. RGWAS is our approach, which uses covariate-aware clusters (MFMR) and tests for genetic variant effect heterogeneity (Het) or globally for any genetic effect (Global). “Previous Subtyping” is like RGWAS, except using covariate-unaware clustering (GMM) and heterogeneity tests. “Inf” means our calculations suffered numerical problems, meaning that λ_*GC*_ is very large. We do not perform SNP tests on NMR PC 5.
(CSV)

S2 Table Statin effect heterogeneity test for *K* ∈ {2, 3, 4} and the 16 traits used to define clusters with MFMR. The “MFMR droptest” columns test using the large-effect covariate test implemented in the rgwas R package and described in the Methods in the main text. The “+Condition on T2D” columns run the same linear model as in droptest, except that T2D status is additionally included as a covariate; this analysis is performed to add confidence that the heterogeneous statin effect on glucose is not merely driven by simple confounding from T2D status.
(CSV)
