# Supplementary material for: Reverse GWAS: Using genetics to identify and model phenotypic subtypes
Source: PLoS Genet. 2019 Apr 5;15(4):e1008009. doi: 10.1371/journal.pgen.1008009 (PMC6469799; doi:10.1371/journal.pgen.1008009)
Supplement: S1 Text — Full description of MFMR model and EM algorithm. Also includes full descriptions of simulations and proofs about PCA/CCA subtype estimators. (PDF) [file pgen.1008009.s001.pdf]

Supplement to Reverse GWAS: Using Genetics to Identify and Model Phenotypic Subtypes

Andy Dahl[1,*], Na Cai[2,3], Arthur Ko[4], Markku Laakso[5,6], Päivi Pajukanta[4], Jonathan Flint[7], and Noah Zaitlen[1,*]

[1]Department of Medicine, UCSF
[2]Wellcome Trust Sanger Institute, Cambridge
[3]European Molecular Biology Laboratory, European Bioinformatics Institute
[4]Department of Human Genetics, David Geffen School of Medicine, UCLA
[5]Institute of Clinical Medicine, Internal Medicine, University of Eastern Finland
[6]Kuopio University Hospital, Kuopio, Finland
[7]Center for Neurobehavioral Genetics, Semel Institute for Neuroscience and Human Behavior, UCLA
[*]andywdahl@gmail.com, noah.zaitlen@ucsf.edu

March 14, 2019

Contents

1 The MFMR model for latent multitrait subtypes **2**

2 EM algorithm to compute the MLE for MFMR **3**
2.1 Quantitative phenotypes 3
2.2 Mixed phenotypes 4
2.3 Computing sufficient statistics with mixed phenotypes 6
2.4 Initialization 9

3 Simulation details **10**
3.1 Ascertaining Case/Control Data 10
3.2 Simulating G-E correlation 11
3.3 Simulating population structure 11

4 Linear contrast subtype estimators **12**
4.1 Linear contrasts for clustering 12
4.2 Testing linear-contrast clusters 13

5 Supplementary Figures and Table **15**

1 The MFMR model for latent multitrait subtypes

We write the standard, univariate interaction model as

$$y_i|Z_{i,}, \alpha, \beta, \sigma^2 \overset{\text{ind}}{\sim} \sum_{q=1}^{Q} X_{iq}\alpha_q + \sum_{s=1}^{S}\sum_{k=1}^{K} \left(G_{is} * Z_{ik}\right)\beta_{ks} + \sum_{k=1}^{K} Z_{ik}\gamma_k + \mathcal{N}\left(0, \sigma^2\right) \tag{1}$$

where:

- $y \in \mathbb{R}^N$ is a single quantitative phenotype.
- Z is an $N \times K$ matrix of subtype weights. In our context, rows of Z are vectors of proportions, meaning they sum to one and have non-negative entries.
- $G \in \mathbb{R}^{N\times S}$ is a matrix of S genotype vectors. Going forward, we include an intercept in G to model the main effect of Z in X and drop the $Z\gamma$ term.
  - Although we primarily seek genetic heterogeneity, G can include any covariates.
  - In practice, only large-effect covariates significantly impact the inferred subtypes, hence we imagine nongenetic variables will be most useful in most human studies.
  - We typically imagine G to have no more than tens of columns.
- X contains all other covariates, which have the same effect regardless of subtype.

This model is equivalent to GxE when Z is interpreted as an environment (though environmental variables do not generally have rows in the probability simplex). An ordinary $G + E$ model is recovered when all the β_k are equal, meaning that the effects of G are identical across subtypes. This can be equivalently parameterized by setting all the β_k to zero and including G in the covariates X, but we find the expression in (1) more readily interpretable. For identification, we implicitly require X and G to have distinct covariates.

(1) can be rewritten for all i as

$$y|Z, \alpha, \beta, \sigma^2 \sim X\alpha + \sum_{s=1}^{S}\sum_{k=1}^{K} \left(G_{,s} * Z_{,k}\right)\beta_{ks} + \mathcal{N}\left(0, \sigma^2 I\right) \tag{2}$$

In (1), $*$ indicated scalar multiplication. In (2), $*$ indicates element-wise multiplication of the vectors $G_{,s}$ and $Z_{,k}$, using $A_{,i}$ to indicate the i-th column of an arbitrary matrix A (likewise, $A_{i,}$ is the i-th row, and $A_{i,,}$ indicates a matrix slice of a three-dimensional array A).

More generally, we define $*$ as the column-wise Khatri-Rao product, which gives a simple expression for interaction between multiple subtypes and genotypes:

$$y|Z, \alpha, \beta, \sigma^2 \sim X\alpha + (G * Z)\beta + \mathcal{N}\left(0, \sigma^2 I\right)$$

Unlike standard interaction tests, we do not assume Z is known. Rather, we assume that each person independently draws one of K random subtypes, i.e. $Z_{i,} = e_k$ for some k, where e_k is a vector of zeros except with a one in entry k. In other words, we assume the group memberships are i.i.d. Categorical with unknown category probabilities p:

$$P(z_i = k|p) = p_k \tag{3}$$

We simply replace α, β and σ^2 by their matrix analogues for multiple traits:

$$Y_{i,}|Z,\alpha,\beta,\Lambda \overset{\text{ind}}{\sim} X_{i,}\alpha + (G * Z)_{i,}\beta + \mathcal{N}\left(0, \Lambda^{-1}\right) \tag{4}$$

We often write $\Sigma = \Lambda^{-1}$. Our generalization to multiple traits implicitly assumes that the clusters Z are common across traits.

2 EM algorithm to compute the MLE for MFMR

We use an expectation-maximization (EM) algorithm to maximize the likelihood of (4) after integrating out z using (3). EM is standard for this type of missing data problem. In our case, EM iterates between an E-step that approximately integrates the likelihood over z (given a current guess for $\theta := \{\alpha, \beta, \Lambda\}$) and an M-step that maximizes the resulting approximate marginal likelihood:

$$Q_t := -\mathbb{E}_{z|\theta^{t-1}}\left(ll(\theta|z,Y)\right) \tag{E-step}$$
$$\theta^t := \arg\max Q_t(\theta) \tag{M-step}$$

In fact, we use an Expectation Conditional-Maximization (ECM) algorithm, conditionally maximizing each block of θ per-E step. ECM has the same guarantee as EM: both converge to a local likelihood maximum [1].

2.1 Quantitative phenotypes

We assume there are no binary traits in this section. We write the matrix Mahalanobis norm w.r.t. Λ as $\|X\|_\Lambda := \text{tr}\left(X\Lambda X^T\right)$; this reduces to the Frobenius norm when $\Lambda = I$ and, more generally, rotates and scales the columns of X before computing the Frobenius norm. The conditional distribution for z_i is Categorical, with the probability of category k given by

$$\begin{aligned} p_{ik}(\theta) := P(z_i = k|Y_{i,},\theta) &\propto P(Y_i|z_i = k, \alpha, \beta, \Lambda)P(z_i = k|p) \\ &\propto \exp\frac{-1}{2}\|Y_{i,} - X_{i,}\alpha - G_{i,}\beta_{k,,}\|_\Lambda^2 \cdot p_k \end{aligned} \tag{5}$$

That is, the probability of cluster k is proportional to the likelihood of $Y_{i,}$ if it were in cluster k, weighted by the overall cluster probability p_k. The E step of standard Gaussian mixture models is obtained by setting $X = 0$ and $G = 1_N$.

We define $p_{ik}^t := p_{ik}(\theta^{t-1})$, where θ^{t-1} are the parameters estimated by the previous EM iteration. The E-step amounts to evaluating these conditional probabilities:

$$\begin{aligned} Q_t &:= -\mathbb{E}_{z|\theta^{t-1}}\left(ll(\alpha,\beta,\Lambda,p|z,Y,G,X)\right) \\ &\equiv -\mathbb{E}_{z|\theta^{t-1}}\left(\log P(Y|\alpha,\beta,\Lambda,z,G,X) + \log P(z|p)\right) \\ &\equiv -\frac{N}{2}\log|\Lambda| + \frac{1}{2}\sum_i\sum_k p_{ik}^t\|Y_{i,} - X_{i,}\alpha - G_{i,}\beta_{k,,}\|_\Lambda^2 - \sum_{i,k} p_{ik}^t \log p_k \end{aligned} \tag{6}$$

We use $\equiv$ to indicate functions with identical optimizers.

The M-step for p just takes the average responsibilities per cluster: $p_k = \frac{1}{N}\sum_i p_{ik}^t$.

The CM-steps for $\beta|\alpha$ immediately split over the K clusters:

$$Q_t(\beta_{k,,}|\alpha) \equiv \sum_i p_{ik}^t \|(Y_{i,} - X_i\alpha) - G_i\beta_{k,,}\|_\Lambda^2$$

This is minimized by regressing the X-residuals–i.e. $Y - X\alpha$–on G while using the weights p_k^t.

Analogously, the α update regresses G-residuals –i.e. $\hat{\delta}_{i,} := Y_{i,} - G\left(\sum_k p_{ik}^t \beta_{k,,}\right)$ –on X:

$$Q_t(\alpha|\beta) \equiv \sum_k \sum_i p_{ik}^t \|\left(Y_{i,} - G\beta_{k,,}\right) - X_i\alpha\|_\Lambda^2 \equiv \text{tr}\left(\left[\sum_i X_{i,}^T X_{i,}\right] \alpha\Lambda\alpha^T - 2\left[\sum_i X_{i,}^T \hat{\delta}_{i,}\right] \Lambda\alpha^T\right) \implies$$
$$\alpha = \left[X^T X\right]^{-1} X^T \hat{\delta}$$

Finally, the Λ update depends on the per-cluster G- and X-residuals $\hat{\epsilon}_{kip} := Y_{ip} - X_{i,}\alpha_{,p} - G_{i,}\beta_{k,p}$. We define $\hat{S}$ as the weighted average of these per-cluster sample covariance matrices:

$$\hat{S} := \sum_k \frac{1}{N} \hat{\epsilon}_{k,,}^T \text{diag}\left(p_{,k}^t\right) \hat{\epsilon}_{k,,} \tag{7}$$

Then the M-step for Λ is

$$Q_t(\Lambda|\hat{\epsilon}) \equiv -N \log|\Lambda| + \text{tr}\left(\hat{S}\Lambda\right) \implies \Lambda^{-1} = \hat{S} \tag{8}$$

We note that all of these updates take extremely simple and common forms. This means that many penalized variants of our likelihood can be trivially solved by appealing to existing, sophisticated third party software in the CM steps (e.g. [2, 3]). Penalizing α or β gives extremely well studied penalized regression CM steps, and penalizing Λ gives penalized precision estimation CM steps.

2.2 Mixed phenotypes

We now allow $Y^b \in \{0,1\}^{N \times B}$, a matrix of B binary phenotypes, in addition to the quantitative phenotypes in $Y \in \mathbb{R}^{N \times P}$. We model the binary phenotypes as truncated versions of latent, quantitative liabilities $Y^l \in \mathbb{R}^{N \times B}$. We then use our quantitative phenotype model on the joined observed and latent quantitative traits $Y' := \left(Y^l : Y\right)$:

$$Y'_{i,}|z_i, \alpha, \beta, \Lambda \overset{\text{ind}}{\sim} \mathcal{N}\left(X_{i,}\alpha + G_{i,}\beta_{z_i,,}, \Lambda^{-1}\right)$$
$$Y_{ip}^b|Y^l = I\{Y_{ip}^l > 0\}$$
$$z_i|p \overset{\text{iid}}{\sim} \text{Categorical}(p)$$

$I\{\cdot\}$ is an indicator function taking value 1 if its argument is true and 0 otherwise.

As is standard in liability threshold models, the parameters $\alpha_{,p}$, $\beta_{,,p}$ and Λ_{pp} are not jointly identified for $p \in \{1, \ldots, B\}$: multiplying these three parameters by any constant gives an equivalent model on Y^b (formally, both $\Lambda_{,p}$ and $\Lambda_{p,}$ have to be scaled by the square root of the constant). So, WLOG, we require $\Lambda_{pp} = 1$ for $p \in \{1, \ldots, B\}$. When only one binary trait is studied, this reduces to the common probit regression constraint that the liability-scale noise has variance 1.

Our EM algorithm now treats both the group memberships z and the latent phenotypes Y^l as missing data. The E-step will require the following sufficient statistics:

$$\begin{aligned} p_{ik} &:= P(z_i = k | Y_{i,}, Y^b_{i,}, \theta) \\ \mu^l_{ik,} &:= \mathbb{E}_{Y^l_{i,}|z_i=k,\theta^t,Y,Y^b}\left(Y^l_{i,}\right) \in \mathbb{R}^B \\ \Sigma^l_{(ik)} &:= \mathbb{V}_{Y^l_{i,}|z_i=k,\theta^t,Y,Y^b}\left(Y^l_{i,}\right) \in \mathbb{R}^{B\times B} \qquad \text{(implicit)} \\ \Sigma^l &:= \frac{1}{N}\sum_{ik} p_{ik}\Sigma^l_{(ik)} \end{aligned}$$

We discuss how to compute these terms in Section 2.3.

Given these sufficient statistic estimates, the Q function can be written

$$\begin{aligned} -Q_t &:= \mathbb{E}_{z,Y^l|\theta^t,Y,Y^b}\left(ll(\theta|Y,z)\right) \\ &\equiv \mathbb{E}_{z,Y^l|\theta^t,Y,Y^b}\left(\log P(Y|z,\alpha,\beta,\Lambda)\right) + \mathbb{E}_{z|\theta^t,Y,Y^b}\left(\log P(z|p)\right) \\ &\equiv \frac{1}{2}\sum_i \mathbb{E}_{z_i,Y^l_{i,}|\theta^t,Y,Y^b}\left(\log|\Lambda| - \|Y'_{i,} - X_{i,}\alpha - G_{i,}\beta_{z_i,,}\|^2_\Lambda\right) + \sum_i\sum_k p_{ik}\log p_k \\ &\equiv N\log|\Lambda| + 2\sum_{i,k} p_{ik}\log p_k \\ &- \sum_i \mathbb{E}_{z_i|\theta^t,Y,Y^b}\left(\|\left(\mathbb{E}_{Y^l_{i,}|z_i,\theta^t,Y,Y^b}\left(Y^l_{i,}\right)\,:\,Y_{i,}\right) - X_{i,}\alpha - G_{i,}\beta_{z_i,,}\|^2_\Lambda - \text{tr}\left(\Lambda\begin{pmatrix} \mathbb{V}_{Y^l_{i,}|z_i,\theta^t,Y,Y^b}\left(Y^l_{i,}\right) & 0 \\ 0 & 0\end{pmatrix}\right)\right) \\ &\equiv N\log|\Lambda| + 2N\sum_k \bar{p}_k\log p_k - \sum_{i,k} p_{ik}\|\left(\mu^l_{ik,}\,:Y_{i,}\right) - X_{i,}\alpha - G_{i,}\beta_{k,,}\|^2_\Lambda - \text{tr}\left(\Lambda_{bb}\Sigma^l\right) \end{aligned}$$

where Λ_{bb} is the block of Λ corresponding to the binary traits.

As the M-step for p and the CM-steps for $\alpha|\beta$ and $\beta|\alpha$ depend only on the first (conditional) moment of Y^l, these steps are identical to the above section, except Y is replaced by the conditional expectation of Y' (see Section 2.3 for details).

The M-step for Λ also involves replacing Y with the conditional expectation of Y' inside a residual, this time to compute the sample covariance $\hat{S}$ (the variance of the expected liabilities). Λ also depends on second moments of Y^l (the expectation of the variance of the liabilities):

$$\hat{S} := \sum_k \frac{1}{N}\hat{\epsilon}^T_{k,,}\text{diag}\left(p^t_{,k}\right)\hat{\epsilon}_{k,,} + \begin{pmatrix} \Sigma^b & 0 \\ 0 & 0 \end{pmatrix}$$

As before, $\hat{S}$ is the weighted average of K sample covariance matrices, but now the sample covariances are penalized to account for uncertainty in Y^l. Using the new $\hat{S}$, equation (8) still provides the Q function for Λ.

Unlike before, it is not true that $\Lambda = \hat{S}^{-1}$ because of the constraint that $\Lambda_{pp} = 1$ for binary traits p. In fact, for computational reasons discussed below, we choose to also constrain $\Lambda_{pq} = 0$ for binary traits $q \neq p$ (when $B = 1$, this constraint is vacuous). Surprisingly, this constrained optimization problem for Λ has a simple, analytic solution, given in Section 2.3, and it turns out that the constrained estimate Λ does not (directly) depend on Σ^b.

2.3 Computing sufficient statistics with mixed phenotypes

The above M steps require the responsibilities, p_{ik}, and the mean and variance of Y^l in each cluster. Without binary traits, the Y^l terms are irrelevant and the p_{ik} have an analytic expression. Otherwise, these quantities all involve potentially complicated Gaussian integrals.

Assume now that $Y^l_{i,}$ is consistent with the sign pattern indicated by $Y^b_{i,}$–otherwise the likelihood is zero. Write the Gaussian density function with covariance matrix Σ and mean 0 evaluated at x given x' by $\phi(x, \Sigma|x')$. Then, the conditional distribution for $Y^l_{i,}|\theta, z_i$, independently for all i, is given by

$$\begin{aligned}
P\left(Y^l_{i,}|\theta, z_i, Y_{i,}, Y^b_{i,}\right) &\equiv P\left(Y^b_{i,}|\theta, z_i, Y_{i,}, Y^l_{i,}\right) P\left(Y^l_{i,}|\theta, z_i, Y_{i,}\right) \\
&\equiv \phi\left(Y'_{i,}, X_{i,}\alpha + G_{i,}\beta_{z_{i},,}, \Lambda|Y_{i,}\right) \cdot I\{Y^l_{i,} \equiv Y^b_{i,l}\} \implies \\
Y^l_{i,}|\theta, z_i = k, Y, Y^b &=: TN_{Y^b_{i,}}\left(\mu^0_{ik,}, \Sigma^0\right) \\
\mu^0_{ik,} &:= X_{i,}\alpha_{,b} + G_{i,}\beta_{k,,b} + \Sigma_{b,q}\left[\Sigma_{q,q}\right]^{-1}\left(Y_{i,} - X_{i,}\alpha_{,q} - G_{i,}\beta_{k,,q}\right)^T \quad (9) \\
\Sigma^0 &:= \left(\Lambda_{b,b}\right)^{-1} \quad (10)
\end{aligned}$$

By TN, we mean a truncated normal distribution supported only on the orthant $\mathcal{O}_{Y^b_{i,}}$, the orthant indicated by the binary values in $Y^b_{i,}$. In particular, this means that

$$\mu_{ik,} := \mathbb{E}\left(Y^l_{i,}|Y^b_{i,}, z_i = k, \theta\right) = \mathbb{E}\left(\mathcal{TN}_{Y^b_{i,}}\left(\mu^0_{ik,}, \Sigma^0\right)\right) \quad (11)$$

$$\Sigma^{ik} := \mathbb{V}\left(Y^l_{i,}|Y^b_{i,}, z_i = k, \theta\right) = \mathbb{V}\left(\mathcal{TN}_{Y^b_{i,}}\left(\mu^0_{ik,}, \Sigma^0\right)\right) \quad (12)$$

$\mu^0_{ik,} \neq \mu_{ik,}$ in general: the former is the expectation for $Y^l_{i,}$ given Y, $z_i = k$ and θ, but not conditional on the orthant indicator. For example, in the case $B = 1$ and $Y^b_i = 1$, it could be that $\mu^0_{ik} < 0$, but $\mu_{ik} < 0$ will never hold because, conditional on Y^b, it is almost certain that Y^l is non-negative. Conversely, if $\mu^0_{ik} \gg 0$ and $Y^b_i = 1$, $\mu^0_{ik} \approx \mu_{ik}$ because conditioning on $Y^l_i > 0$ adds little information and thus has minimal impact on the expectation.

z_i is still Categorical, but now the analogue of (5) integrates $Y^l_{i,}$ over its domain $\mathcal{O}_{Y^b_{i,}}$:

$$\begin{aligned}
p_{ik}(\theta) :&= P(z_i = k|Y_{i,}, Y^b_{i,}, \theta) \\
&\propto P(Y_{i,}, Y^b_{i,}|\theta)P(z_i = k|\theta) \\
&= \int_{Y^l_{i,} \in \mathcal{O}_{Y^b_{i,}}} P(Y_{i,}|z_i = k, \theta)P(Y^l_{i,}|Y_{i,}, z_i = k, \theta)p_k \\
&\propto p_k P(Y_{i,}|z_i = k, \theta) \int_{Y^l_i \in \mathcal{O}_{Y^b_{i,}}} P(Y^l_{i,}|Y_i, z_i = k, \theta) \\
&\equiv \underbrace{p_k}_{\text{Prior}} \times \underbrace{\phi\left(Y_{i,}, X_{i,}\alpha_{,-b} + G_{i,}\beta_{k,,-b}, \Sigma_{-b,-b}\right)}_{\text{Quantitative Likelihood}} \times \underbrace{\int_{Y^l_i \in \mathcal{O}_{Y^b_{i,}}} \phi\left(Y^l_{i,}, \mu^0_{ik,}, \Sigma^0\right)}_{\text{(Conditional) Binary Likelihood}}
\end{aligned}$$

The first two terms capture Y but essentially ignore Y^b, giving simple expressions essentially identical to those for the quantitative-trait-only p_{ik} (equation (5)). The third term is a (multivariate) probit likelihood for Y^b given Y and cannot easily be evaluated in general.

Only one binary phenotype

When $B = 1$, the orthant $\mathcal{O}_{Y^b_{i,}}$ simplifies to one half of the real line and the covariance matrix $\Sigma^0 \in \mathbb{R}^{1\times 1}$ is equal to 1 WLOG, and the complicated third term in the responsibilities reduces to a standard Gaussian c.d.f. evaluation (Φ):

$$\int_{Y^l_i \in \mathcal{O}_{Y^b_{i,}}} \phi\left(Y^l_{i,}, \mu^0_{ik,}, \Sigma^0\right) = \left\{ \begin{array}{cc} \Phi\left(-\mu^0_{ik}\right) & \text{if } Y^b_i = 0 \\ 1 - \Phi\left(-\mu^0_{ik}\right) & \text{if } Y^b_i = 1 \end{array} \right\} =: \tilde{\Phi}_{b_i}\left(-\mu^0_{ik}\right)$$

where $\tilde{\Phi}_{\cdot}$ is either the lower or upper tail probability and $b_i = Y^b_i$ is a vector of disease indicators. The value of this representation is that the standard normal c.d.f. Φ has been numerically tabulated.

Similarly, the complicated multidimensional truncated normal distributions on Y^l become univariate truncated normals. This dramatically simplifies computation because the univariate truncated normal has analytic mean and variance formulas. First, let

$$\psi_b(z) = \frac{\tilde{\phi}_b\left(z\right)}{\tilde{\Phi}_b\left(z\right)}$$

where $\tilde{\phi}_b = \tilde{\Phi}'_b$ and is either the standard Gaussian pdf if $b = 0$–which we call ψ, defined as $\psi(\cdot) = \phi(\cdot, 1)$–or $-\psi$. Then, the sufficient statistics can be written:

$$\mu_{ik} := \mathbb{E}\left(\mathcal{TN}_{b_i}\left(\mu^0_{ik}, 1\right)\right) = \mu^0_{ik} - \psi_{b_i}\left(-\mu_{ik}\right) \quad (13)$$

$$\Sigma^{ik} := \mathbb{V}\left(\mathcal{TN}_{b_i}\left(\mu^0_{ik,}, 1\right)\right) = 1 + \mu_{ik}\psi_{b_i}\left(-\mu_{ik}\right) - \psi_{b_i}\left(-\mu_{ik}\right)^2 \quad (14)$$

These terms split over i and k and are easy to compute.

Conditionally independent binary traits

A similar argument for $B = 1$ can be extended to $B > 1$ latent traits that are conditionally independent, i.e. in the setting where $\Sigma^0 = \left(\Lambda_b\right)^{-1}$ is diagonal or, equivalently because diagonal entries of Λ_b are 1, where $\Sigma^0 = I_B$. This conditional independence parameterization requires that all correlation between binary traits be captured in α, β, and noise correlations with quantitative traits. In other words, our model can express systematic misdiagnosis patterns but will struggle in situations where doctors choose between mutually exclusive diagnoses more or less at random.

In the METSIM analysis, we encoded pre-type 2 diabetes as a binary indicator that also takes value 1 for type 2 diabetics, which makes the conditional independence assumption much more viable:. Otherwise, there is a mutual exclusivity between columns of Y^b that cannot be expressed except through covariates that have distinct effects–rather than graded effects–on pre-T2D and T2D. This choice made our model fit substantially more interpretable, and related choices are important for any analyses with ordinal categories.

Regardless of the plausibility, we can maximize our likelihood subject to the constraint that $\Lambda_{ij} = \Lambda_{ji} = 0$ whenever i and j index distinct, binary traits. Under this assumption, the conditional expectations and variances in (11) and (12) simplify:

$$\mu_{ikp} := \mathbb{E}\left(\mathcal{TN}_{Y^b_{ip}}\left(\mu^0_{ikp}, 1\right)\right) = \mu^0_{ikp} - \psi_{Y^b_{ip}}\left(-\mu^0_{ikp}\right)$$

$$\Sigma^{ik} := \mathbb{V}\left(\mathcal{TN}_{b_i}\left(\mu^0_{ik,}, 1\right)\right) = 1 + \mu^0_{ikp}\psi_{Y^b_{ip}}\left(-\mu_{ikp}\right) - \psi_{Y^b_{ip}}\left(-\mu_{ikp}\right)^2$$

M-step details with mixed phenotypes: α and β

As the M-step for p and the CM-steps for $\alpha|\beta$ and $\beta|\alpha$ depend only on the first (conditional) moment of Y^l, these steps are identical to the above section, except Y is replaced by the conditional expectation of Y'. The only detail is that the expectation of Y' now depends on k, unlike Y.

For α, the update is

$$\begin{aligned} Q_t(\alpha|\beta) &\equiv \sum_k \sum_i p_{ik}^t \| ((\mu_{ik,} : Y_{i,}) - G_{i,}\beta_{k,,}) - X_{i,}\alpha\|_\Lambda^2 \\ &\equiv \sum_i \left(X_{i,}\alpha\Lambda\alpha^T X_{i,}^T - 2 \left[\underbrace{\sum_k p_{ik} \left[(\mu_{ik,} : Y_{i,}) - G\beta_{k,,} \right]}_{\hat{\delta}_{i,}} \right] \Lambda\alpha^T X_{i,}^T \right) \implies \\ \alpha &= \left[X^T X \right]^{-1} X^T \hat{\delta} \end{aligned}$$

Similarly, β is again WLS, but the first B columns of Y are now the expected liabilities:

$$\begin{aligned} Q_t(\beta_{,,k}|\alpha) &\equiv \sum_i p_{ik}^t \| \left(\underbrace{(\mu_{ik,} : Y_{i,}) - X_i\alpha}_{\hat{\epsilon}_{i,}} \right) - G_{i,}\beta_{k,,}\|_\Lambda^2 \implies \\ \alpha &= \left[G^T \mathrm{diag}\left(p_{,k}\right) G \right]^{-1} G^T \mathrm{diag}\left(p_{,k}\right) \hat{\epsilon} \end{aligned}$$

M-step details with mixed phenotypes: Λ

As a function of Λ, Q is given by (8). By assumption, we can write Λ in block matrix form as:

$$\Lambda = \begin{pmatrix} I_B & C^T \\ C & D \end{pmatrix}$$

Applying Sylvester's determinant lemma simplifies the log-determinant term in (8):

$$\log|\Lambda| = \log \left| \begin{pmatrix} I & C^T \\ C & D \end{pmatrix} \right| = \log|I| + \log\left|D - CI^{-1}C^T\right| \equiv \log\left|D - CC^T\right|$$

The trace term is even simpler:

$$\mathrm{tr}\left(\Lambda\hat{S}\right) = \mathrm{tr}\left(\hat{S}\begin{pmatrix} I & C^T \\ C & D \end{pmatrix}\right) \equiv \mathrm{tr}\left(\hat{S}_{qq}D\right) + 2\mathrm{tr}\left(\hat{S}_{qb}C\right)$$

using $\hat{S}_{qq}$ to denote the $(P-B)\times(P-B)$ submatrix of $\hat{S}$ corresponding to the quantitative traits (and analogously for $\hat{S}_{qb}$).

Optimizing over the parameters C and D gives the first order conditions:

$$
\begin{aligned}
\nabla_D Q(\Lambda|\hat{S}) &= \left(D - CC^T\right)^{-1} = \hat{S}_{qq} \\
\nabla_C Q(\Lambda|\hat{S}) &= -\left(D - CC^T\right)^{-1} C = \hat{S}_{qb} \\
&\implies \\
(C, D) &= \left(-\left[\hat{S}_{qq}\right]^{-1} \hat{S}_{qb}, \left[\hat{S}_{qq}\right]^{-1} + \left[\hat{S}_{qq}\right]^{-1} \hat{S}_{qb}\hat{S}_{qb}^T \left[\hat{S}_{qq}\right]^{-1}\right)
\end{aligned}
$$

To our knowledge, this idea for easing multivariate probit computation is novel. The main approach we are aware of is to numerically integrate the B-dimensional Gaussian integrals appearing for the probit terms, which does not scale to B above 4 (or, being very generous, 10). By contrast, our approach scales to tens of traits. The cost, of course, is the conditional independence assumption, which becomes increasingly stringent as B increases (in the sense that $B(B-1)$ entries of the precision Λ are forced to 0).

Log-likelihood

The likelihood is

$$
\begin{aligned}
L(\theta|Y_{i,}) &= \int_{Y_i^l \in \mathcal{O}_{Y_i^b}} P(Y_{i,}|\theta) dY_{i,}^l \\
&= \sum_k p_k P(Y_{i,}|\theta, z_i = k) \int_{Y_{i,}^l \in \mathcal{O}_{Y_{i,}^b}} P(Y_{i,}^l|Y_{i,}, \theta, z_i = k) dY_{i,}^l \\
&= \sum_k p_k \phi(Y_{i,}|X\alpha_{,-b} + G\beta_{k,,-b}, \Sigma_{-b,-b}) \int_{Y_{i,}^l \in \mathcal{O}_{Y_{i,}^b}} \phi(Y_{i,}^l|\mu_{ik,}^0 \Sigma^0) dY_{i,}^l
\end{aligned}
$$

where μ^0 and Σ^0 are defined by α, β and Λ by (9) and (10).

By assumption on Σ^0, the integral over $Y_{i,}^l$ breaks into the product of B univariate Gaussian integrals. Letting $\mathcal{B}$ be the (potentially empty) set of binary traits, the full log-likelihood is

$$
\ell(\theta|Y) \equiv \sum_i \log\left(\sum_k p_k \left(|\Sigma_{-\mathcal{B},-\mathcal{B}}|^{-1/2} \exp \frac{1}{2}\|Y_{i,} - X_i\alpha - G_i\beta_k\|^2_{\Sigma_{-\mathcal{B},-\mathcal{B}}}\right) \times \prod_{p\in\mathcal{B}} \tilde{\Phi}_{Y_{ip}^b}\left(-\frac{\mu_{ikp}^0}{\sqrt{\Sigma_{pp}^0}}\right)\right)
$$

To mitigate numerical underflow, we compute these products of exponentials as sums of their logarithms and exponentiate at the end.

2.4 Initialization

By default, we initialize by setting $\alpha = \beta = 0$ and Λ^{-1} equal to the sample trait covariance, imposing constraints on Λ_b if $B > 1$. Then, we set the slice of β corresponding to main subtype effects to i.i.d. Gaussians with standard deviation 10^{-2} (we scale quantitative traits to mean 0, variance 1). In simple settings, including all our simulations, a single initialization suffices.

Real data is more complex, however, and in practice we perform 10 random initializations and choose the run obtaining the highest likelihood. This was unnecessary in CONVERGE but valuable in METSIM, as different initalization reaches different modes. Generally, we see restarts as mitigating the impact of very poor local optima rather than as guaranteeing the global optimum.

3 Simulation details

Many of our simulations begin by drawing a dataset from the MFMR model in (4) and (3). This model is parameterized by homogeneous effects (α), heterogeneous effects (β), a noise precision matrix (Λ), and cluster sizes (p). We generate the covariates to resemble SNPs (see below), but otherwise they have no genetic meaning (but see Section 3.3). We take $P = 30$ traits and make three binary by thresholding. By default, we set $K = 2$ and $p = (0.7, 0.3)$, to represent subtypes with unequal prevalence.

We draw 12 columns for G, independently, by drawing an allele frequency by $\pi \sim \text{Uniform}[0.05, 0.5]$ and then drawing Binomial(2,π) SNP genotypes for each sample.

We link these genotypes to the phenotypes by assuming 4 have no effect (S_{null}), 4 have homogeneous effects (S_{hom}), and 4 have heterogeneous effects (S_{het}). By default, SNPs have the same type of effect on all traits. To parameterize this mixture distribution on effect sizes, we draw the array of coefficients $\beta \in \mathbb{R}^{K\times 12\times P}$ for each SNP s and phenotype p by

$$\beta_{,sp} \overset{\text{ind}}{\sim} \begin{cases} (0\ 0) & \text{if SNP } s \text{ is null} \\ \mathcal{N}\left(0, \frac{1}{S_{hom}}\sigma^2_{hom}\right)\cdot(1,1) & \text{if SNP } s \text{ is hom.} \\ \mathcal{N}\left(0, \frac{1}{.7\cdot S_{het}}\sigma^2_{hom}\right)\cdot(1,0) & \text{if SNP } s \text{ is het.} \end{cases} \tag{15}$$

In this stylized model, genetic effects are either homogeneous or active only in group 1. This is inspired by the CONVERGE dataset, where the three heterogeneous SNPs appeared to have no effect in the smaller, high-stress group.

We add large main subtype effects $\mu_{kp} \overset{\text{iid}}{\sim} \mathcal{N}\left(0, \sigma^2_z = .1\right)$ to trait p for all samples in group k.

For $K > 2$ (for Supplementary Figure 4b), we drop the asymmetry between groups. For group sizes, this means we set $p = \frac{1}{K}1_K$. We also now draw the heterogeneous covariates by

$$\beta_{,sp} \overset{\text{ind}}{\sim} \begin{cases} 0_K & \text{if SNP } s \text{ is null} \\ 1_K\mathcal{N}\left(0, \frac{1}{S_{hom}}\sigma^2_{hom}\right) & \text{if SNP } s \text{ is hom.} \\ \mathcal{N}\left(0_K, \frac{1}{S_{het}}\sigma^2_{het}I_K\right) & \text{if SNP } s \text{ is het.} \end{cases} \tag{16}$$

By default, $\sigma^2_{hom} = 0.04$ or 0.004, which represent very large SNP effects or modest covariate effects. We take $\sigma^2_{het} = 0.044 - \sigma^2_{hom}$ so that the total heritability stays constant when σ^2_{hom} changes.

Finally, we draw $\Sigma_0 \sim \text{Wi}(P, I_P)$ and then take $\Lambda^{-1} = \left(1 - \sigma^2_z - \sigma^2_{hom} - \sigma^2_{het}\right)\texttt{cov2cor}(\Sigma_0)$, where `cov2cor` scales the columns and rows of a covariance matrix to give its corresponding correlation matrix. This implies the σ^2 terms can be interpreted as fractions of variance explained.

3.1 Ascertaining Case/Control Data

A common feature of real association studies is case ascertainment, where diseased samples are preferentially ascertained so that the case/control ratio is roughly balanced. This is particularly important for rare diseases, as random sampling would obtain very few disease examples.

We model this process by generating $100\times N$ samples from our above model and then selecting $\frac{N}{2}$ cases and controls (uniformly at random). This is computationally wasteful as $99\times N$ samples are generated only to be discarded. This waste can be eliminated in the case of a single phenotype [4], which can likely be generalized to multiple traits.

As a result of the ascertainment process, the simulated data no longer exactly match our model in (4). In particular, the so-called oracle is no longer a true oracle, explaining the false positive inflation for Hom SNPs. But in a model-false world [5], the relevant question is approximate calibration, and a massive amount of data is required to falsify the null model of SNP homogeneity in the Case/Control simulations when using the oracle (or MFMR) z. By constrast, GMM returns false positives across the spectrum of N.

3.2 Simulating G-E correlation

We modify our baseline simulation so that the subtype state z is correlated with the SNP genotypes in G. We do this by first drawing a continuous proxy for the subtypes, $\tilde{z}$, by:

$$\begin{aligned} \tilde{z} &= \sqrt{\rho_{GE}}\, G\omega + \sqrt{1-\rho_{GE}}\,\delta \\ \omega_i &\overset{\text{iid}}{\sim} \mathcal{N}\left(0, 1/S\right) && (S{=}\#\ \text{SNPs}) \\ \delta_i &\overset{\text{iid}}{\sim} \mathcal{N}\left(0, 1\right) \end{aligned}$$

As ρ_{GE} increases from 0 to 1, $\tilde{z}$ goes from completely independent of genotype (as in our baseline simulation) to completely heritable ($|\rho_{GE}|$ is the heritability of $\tilde{z}$).

We then threshold this continuous subtype axis to recover discrete subtypes by setting $z_i = 1$ if $\tilde{z}_i > \tau$ and $z_i = 0$ otherwise. Here, τ is chosen to ensure the appropriate subtype prevalences.

3.3 Simulating population structure

For each simulated dataset, we drew 10,000 SNPs independently as follows. First, we set an ancestral allele frequency of 50%. Second, we independently drew two population allele frequencies, p_i, from a Beta distribution with mean 0.5 and variance $F_{st} = 0.5(1-0.5)$, choosing $F_{st} = 0.1$. Finally, we draw $N/2$ Binomial$(2, p_i)$ SNPs for $i = 1, 2$.

We estimate genetic PCs from these 10,000 SNPs, but only the 12 we randomly choose to use in the simulation have any effect or are tested.

We create population structure by adding population main effects with variance σ^2_{pop}. We draw these effects independently from mean zero Gaussian distributions for each population and trait. We also modify the environmental variance, for all traits, to $\sigma_e^2 := 1 - \sigma_z^2 - \sigma^2_{hom} - \sigma^2_{het} - \sigma^2_{pop}$. Again, this means the σ^2 terms can be interpreted as proportions of explained variance.

4 Linear contrast subtype estimators

CCA seems to outperform the oracle in some simulations, e.g. with very strong main subtype effects (Supplementary Figure 2c) yet seems to have no ability to learn z (Supplementary Figure 1). This apparent discrepancy is partially explained by the simulations in 3, as the CCA positive rate depends little on the true interaction effect for the tested trait.

In this section, we give a theoretical argument, under simplifying assumptions, that bolsters these conclusions from the simulation. In 4.1, we introduce a simplified version of the MFMR model and discuss estimators of $\hat{z}$ built as a linear combination of the phenotypes. In 4.2 we approximate the interaction estimates when using such $\hat{z}$ estimates, conditional on the generating linear transformation, and derive the bias when only some traits have true heterogeneity.

4.1 Linear contrasts for clustering

Let $Y \in \mathbb{R}^{N \times P}$ be a phenotype matrix. Assume there are $K = 2$ true clusters defined by an indicator variable z. The "linear" methods to estimate z that we discuss in this section are all defined by some contrast vector $v \in \mathbb{R}^P$:

$$\hat{z} := Yv \in \mathbb{R}^N \tag{17}$$

In practice, v will often be constructed directly from Y, e.g. it may be the top PC of Y. We ignore this, however, and assume v is defined *a priori*. (More general, tedious calculations like those in [6] could be pursued in some special cases, like when v is a PC.)

In this section, we think of z as a vector in $\mathbb{R}^N$ with two unique entries, normalized to length one and mean zero. Embedding z like this is natural, at least for $K = 2$, when estimating with continuous-valued $\hat{z}$ (and column-centered Y). We assume a simplified version of our general model, ignoring covariates and using only a single SNP $g \in \mathbb{R}^N$ (normalized to length 1 and mean zero):

$$Y = z\alpha^T + (g * z)\gamma^T + \epsilon$$

where ϵ has i.i.d. Gaussian entries with variance σ^2 and α and γ are the subtype main- and interaction-effects. We omit noise correlation and other covariates and require that Y (and X and z) are column-demeaned. We omit main effects for g to simplify calculations and notation later.

Approximating Y as random and v as fixed, the expected fitted clusters are

$$\mathbb{E}(\hat{z}) = \mathbb{E}(Y)\, v = \left(z\alpha^T + (g * z)\gamma^T\right) v = z(\alpha^T v) + (g * z)(\gamma^T v) \tag{18}$$

This decomposes the mean $\hat{z}$ into a combination of z and $g * z$ and immediately suggests:

- If $\alpha = 0$ and g is independent of z, $\mathbb{E}(\hat{z})$ is uncorrelated with z. Intuitively, linear clustering fails without a main subtype effect.
- $\mathrm{Cor}(z, \mathbb{E}(\hat{z}))$ improves as v tags the main effect more and the interaction effect less.

4.2 Testing linear-contrast clusters

Rather than ask whether linear contrasts give good estimates of z, we now ask whether they give good genetic heterogeneity regression coefficients. First, some notation:

$$\begin{aligned}
\hat{z} &:= Yv = z(\alpha^T v) + (g * z)(\gamma^T v) + \epsilon v \\
&:= az + b(g * z) + e \\
x &:= \hat{z} * g \\
X &:= \begin{pmatrix} \hat{z} & g \end{pmatrix} \\
\rho &:= g^T \hat{z}
\end{aligned}$$

Writing P_X as the projection onto X, the regression coefficient for y on x given g and $\hat{z}$ is

$$\hat{\beta} := \frac{x^T (I - P_X) y}{x^T (I - P_X) x}$$

Now we simplify the projections:

$$\begin{aligned}
\left(X^T X\right)^{-1} = \begin{pmatrix} 1 & \hat{z}^T g \\ \hat{z}^T g & 1 \end{pmatrix}^{-1} &= \frac{1}{1-\rho^2} \begin{pmatrix} 1 & -\rho \\ -\rho & 1 \end{pmatrix} \implies \\
P_X = \frac{1}{1-\rho^2} X \begin{pmatrix} 1 & -\rho \\ -\rho & 1 \end{pmatrix} X^T &= \frac{1}{1-\rho^2} \left(\hat{z}\hat{z}^T + gg^T - \rho g \hat{z}^T - \rho \hat{z} g^T \right) \implies \\
x^T P_X x &= \frac{1}{1-\rho^2} \left([x^T \hat{z}]^2 + [x^T g]^2 - 2\rho [x^T \hat{z}][x^T g] \right) \\
x^T P_X y &= \frac{1}{1-\rho^2} \left([x^T \hat{z}][y^T \hat{z}] + [x^T g][y^T g] - \rho [x^T \hat{z}][y^T g] - \rho [y^T \hat{z}][x^T g] \right)
\end{aligned}$$

Though these expressions are messy, most terms will vanish for large N.

All these inner products of interactions can be written as the expectation of polynomials in the independent variables ϵ, g and z, where the expectation is over the empirical distribution. E.g.

$$\begin{aligned}
x^T g &= [(az + b(g * z) + e) * g]^T g = \sum_i \left((az_i + b(g_i * z_i) + e_i) * g_i \right) g_i = \sum_i \left(az_i g_i^2 + bz_i g_i^3 + e_i g_i^2 \right) \\
&= \hat{\mathbb{E}} \left(azg^2 + bzg^3 + eg^2 \right)
\end{aligned}$$

where, abusing notation, z, g and e are understood as empircally-distributed, scalar random variables inside the empirical distribution expectation operator $\hat{\mathbb{E}}$.

For large N , these expectations converge to their population counterparts ($\hat{\mathbb{E}}(\cdot) \to \mathbb{E}(\cdot)$ if (z, g, e) converges weakly in distribution). Since z, g and e are independent and mean zero, expectations of polynomials with any odd-powered terms disappear. Similarly, even-powered expectations converge to variance/kurtosis terms, e.g.

$$\begin{aligned}
x^T \hat{z} &\to \mathbb{E} \left(g \left(az + bg \cdot z + e \right)^2 \right) = 2ab \\
\rho &\to \mathbb{E} \left(g \left(az + bg \cdot z + e \right) \right) = 0 \\
&\implies \\
x^T P_X x &\approx \frac{1}{1-\rho^2} \left(x^T \hat{z} \right)^2 \to 4a^2 b^2
\end{aligned}$$

(The approximation drops some terms that converge to zero for large N. Again, the goal is only to qualitatively explain Supplementary Figure 3.)

To finish computing the asymptotic regression coefficient, we assume (WLOG) we are testing the first trait and that this trait has a nonzero main subtype effect but zero genetic heterogeneity. (I.e. $y = Y_{,1} = z\alpha_1 + \epsilon_{,1}$). Then:

$$y^T\hat{z} = (z\alpha_1 + \epsilon_{,1})^T(az + b(g * z) + e) \to \alpha_1 a + v_1\sigma^2 \implies$$
$$x^T P_X y \approx \frac{1}{1-\rho^2}\left([x^T\hat{z}][y^T\hat{z}]\right) \to 2ab\left(\alpha_1 a + v_1\sigma^2\right) \implies$$
$$\hat{\beta} = \frac{x^T(I - P_X)y}{x^T(I - P_X)x} \to \frac{x^T y - 2ab\left(\alpha_1 a + v_1\sigma^2\right)}{x^T x - 4a^2b^2}$$

$\hat{\beta}$ is pure bias because y has no genetic heterogeneity (by assumption).

The unconditional regression terms, $x^T y$ and $x^T x$, converge to

$$x^T y \to \mathbb{E}\left(g(az + bgz + e)\left[z\alpha_1 + \epsilon_{,1}\right]\right) = \alpha_1 b$$
$$x^T x \to \mathbb{E}\left(g^2(az + bgz + e)^2\right) = a^2 + b^2\nu_g + \sigma^2$$

where ν_g is the kurtosis of g.

Altogether, this gives the asymptotic bias

$$\hat{\beta} \to b\frac{\alpha_1(1 - 2a^2) - 2av_1\sigma^2}{a^2 + b^2\nu_g - 4a^2b^2 + \sigma^2}$$

We make two basic observations about about this large-sample Gx$\hat{z}$ bias:

- The bias is zero if $b = 0$, which necessarily holds if all genetic heterogeneity is absent ($\beta = 0$)
- Otherwise, the bias is nonzero unless v is orthogonal to γ. This will not hold for e.g. PCA.
  - Even as N grows large and clustering works well (in the senses that $\hat{z} \approx z$ and v is roughly proportional to α), b does not converge to 0 unless γ and α are roughly orthogonal (there is no obvious reason why this would ever happen).
- The bias persists even if there is no main subtype effect on y (i.e. $\alpha_1 = 0$).
  - This is a conditioning bias in the sense that it disappears if $\hat{z}$ is not used as a covariate.
  - Also, it is an overfitting bias in that it disappears if $v_1 = 0$, i.e. the weight of the first phenotype in defining $\hat{z}$ is zero. This $v_1 = 0$ condition is satisfied by holding out $Y_{,1}$ when defining $\hat{z}$ or when P grows large (and entries of v are $O(P^{-1})$). However, these steps are only sufficient because we assumed columns of ϵ are uncorrelated.

5 Supplementary Figures and Table

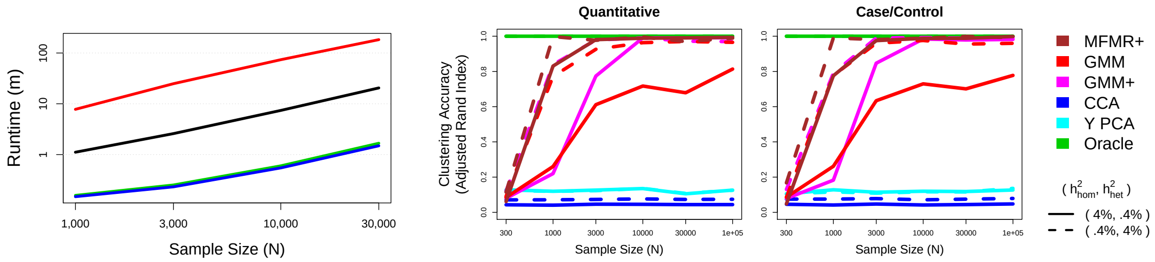

Figure 1: **Running time and subtype estimation accuracy in simulations.** Left: Average running times in main Figure 1 (excluding failed GMM runs). Right: Clustering accuracy for simulations without (‘Quantitative’, as in main Figure 1) and with ascertainment (‘Case/Control’, as in Supplementary Figure 2). We measure accuracy with adjusted Rand index, which varies from 0 (random guessing) to 1 (exact match). We compute the index only across pairs from a random 300 subsamples, reducing computation roughly $\approx 10^5$-fold when $N = 100,000$. Accuracies are estimated for roughly 300 simulations per point in the plot. MFMR+ is shown for simplicity because MFMR gives different clusters per tested SNP.

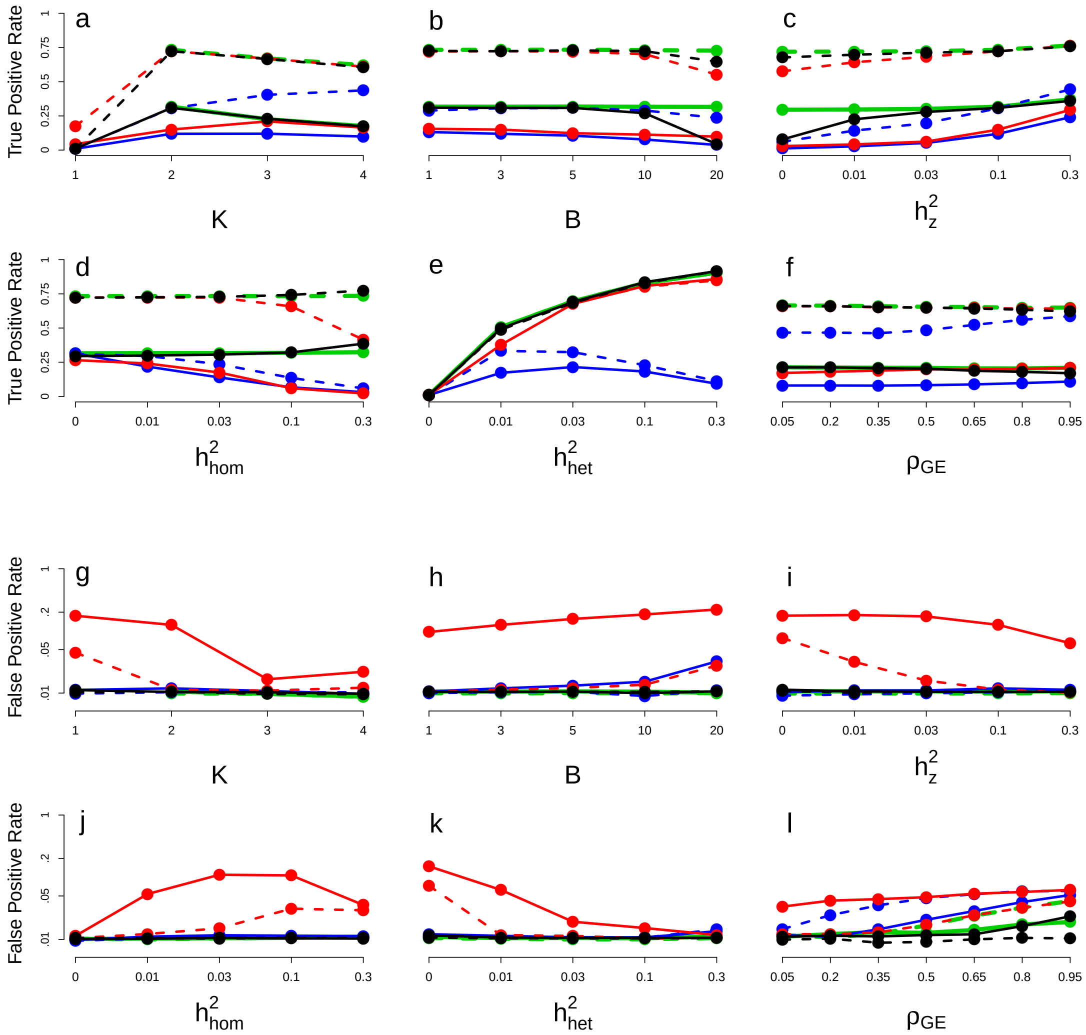

Figure 2: **Simulations varying several further parameters.** Tests for truly heterogeneous SNPs are shown in the top 6 panels (a-f), and the corresponding tests for SNPs with only homogeneoues effects are shown in the below 6 panels (g-l). K is the number of true, simulated subtypes and B is the number of binary traits. ρ_{GE} is the gene-subtype correlation term, with $\rho_{GE} = 0$ giving non-heritable subtype statuses and $\rho_{GE} = 1$ giving perfectly heritable subtypes. h^2_{hom}, h^2_{het}, and h^2_z are the variances explained by homogeneous SNPs, heterogeneous SNPs, and main subtype effects, respectively. As in main Figure 1, solid lines have $(h^2_{hom}, h^2_{het}) = (4\%, .4\%)$, and dashed lines are reversed; in (d,e), line types define only the h^2 term not governed by the x-axis. In (a), all methods fit $K = 2$ subtypes; there is no true heterogeneity for $K = 1$, where the oracle is not defined, and for $K > 1$ and the oracle picks a true cluster at random. Generally, increasing the heterogeneous factors (h^2_{het} and h^2_z) makes subtyping easier, while increasing h^2_{hom} makes subtyping harder.

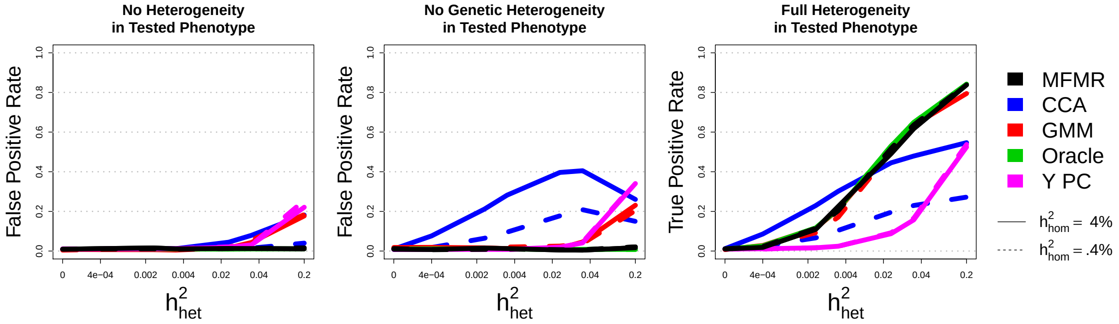

Figure 3: **Simulations where SNP heterogeneity only exists for some traits**, for which they are only homogeneous. Left: the tested trait has no genetic heterogeneity or main subtype effect. Center: the tested trait has only a main subtype effect but no heterogeneity. Right: the full heterogeneity simulation. Linear subtype estimators (CCA and Y PC) are not trait-specific.

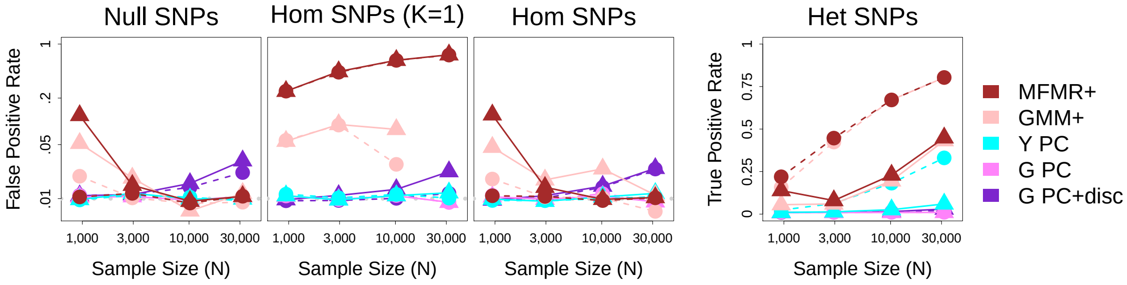

Figure 4: **Main Figure 1 with further subtyping methods.** MFMR+ varies MFMR by treating the tested SNP as heterogeneous. GMM+ varies GMM by including the SNPs as traits when clustering. As expected, MFMR+ and GMM+ are miscalibrated. GMM+ often fails to converge, especially for $N \geq 10,000$ (we evaluate only the converged runs). The other methods, with low power, define subtypes as the top PC of Y or G, optionally thresholded to be binary ("G PC+disc").

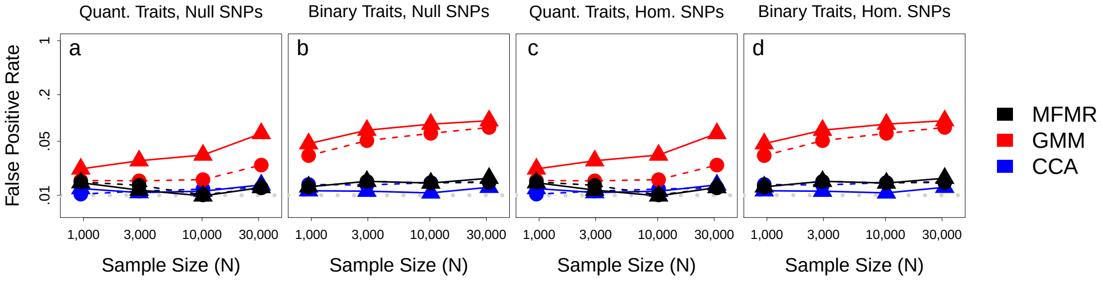

Figure 5: **Simulations with non-Gaussian noise.** Purely homogeneoues simulations, without subtypes, where the noise, ϵ, has marginal t_5 distributions. ϵ is simulated by drawing i.i.d. t_5-distributed random variables, arranging into an $N \times P$ matrix, and then right-multiplying with $\Sigma^{1/2}$, where Σ is the noise covariance matrix and is drawn as in the main simulations in main Figure 1.

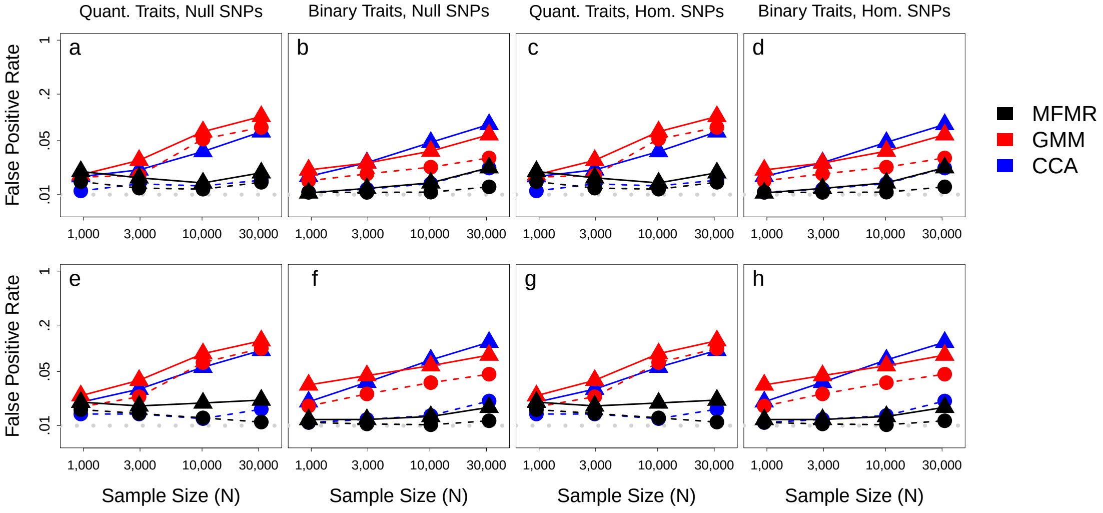

Figure 6: **Simulations with non-linear homogeneous effects.** Purely homogeneoues simulations, without subtypes, where SNPs truly have a non-linear effect. In (a-d), the SNPs are squared before use in MFMR, so that the true SNP and the utilized covariate (i.e. SNP^2) have zero correlation. In (e-h), the true SNPs are exponentiated before inclusion in MFMR, so the true SNP effect is log-linear. Results are partitioned by whether the tested traits are quantitative or binary, as well as by whether the true SNP effect is null or homogeneous.

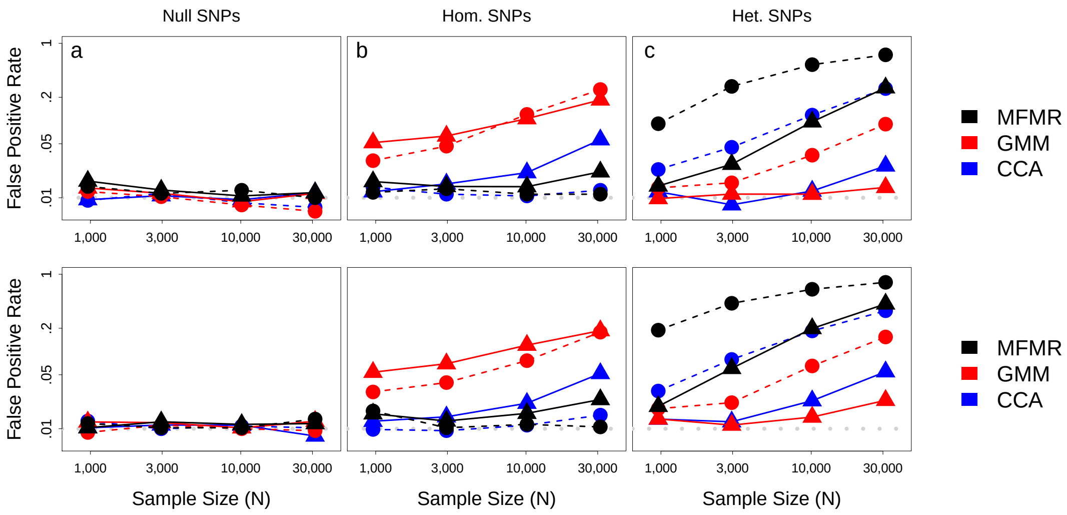

Figure 7: **Simulations with continuously-varying subtypes.** z is chosen to be Gaussian. Top: Effect sizes are chosen so that power roughly matches main Figure 1; it is not trivial to directly convert effect sizes from the discrete z simulations. Bottom: All heterogeneoues effect sizes are doubled relative to top panels.

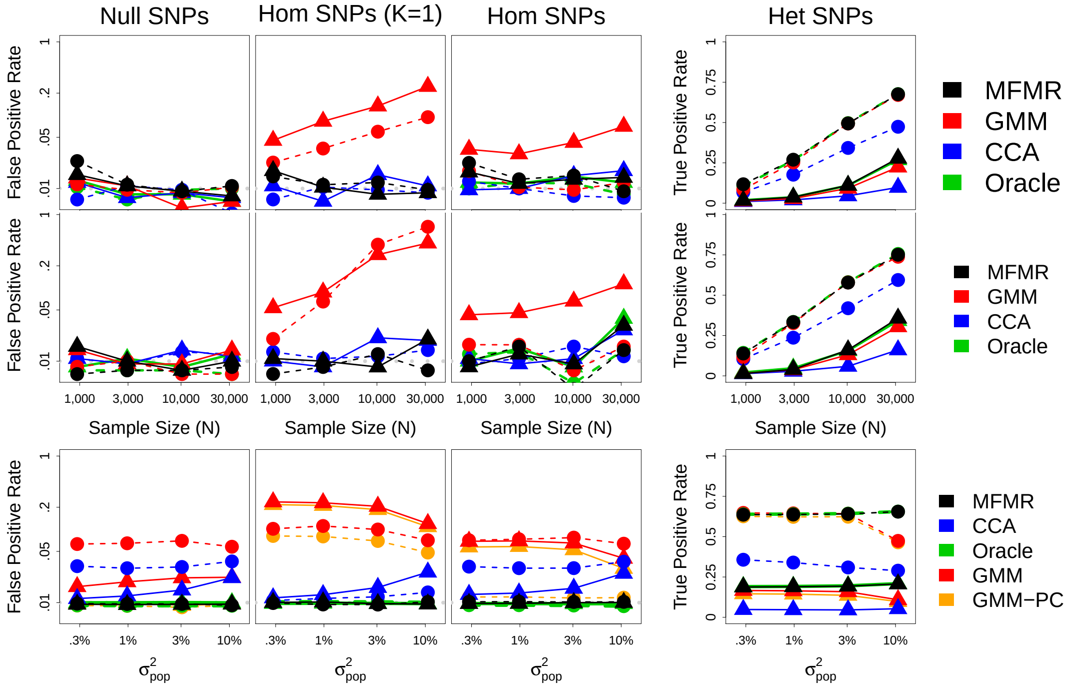

Figure 8: **Alternate versions of Figure 1**. Top: SNP effect heterogeneity tests are applied to binary traits, not quantitative traits as in main Figure 1. Even though GMM only clusters the quantitative traits, tests for the (correlated) binary traits are miscalibrated. Middle: a 20% population prevalence binary trait is ascertained to have 50% in-sample prevalence and then tested. Bottom: population structure is added and MFMR, Oracle and GMM-PC test conditional on three genetic PCs; GMM and GMM-PC use the same subtype estimator.

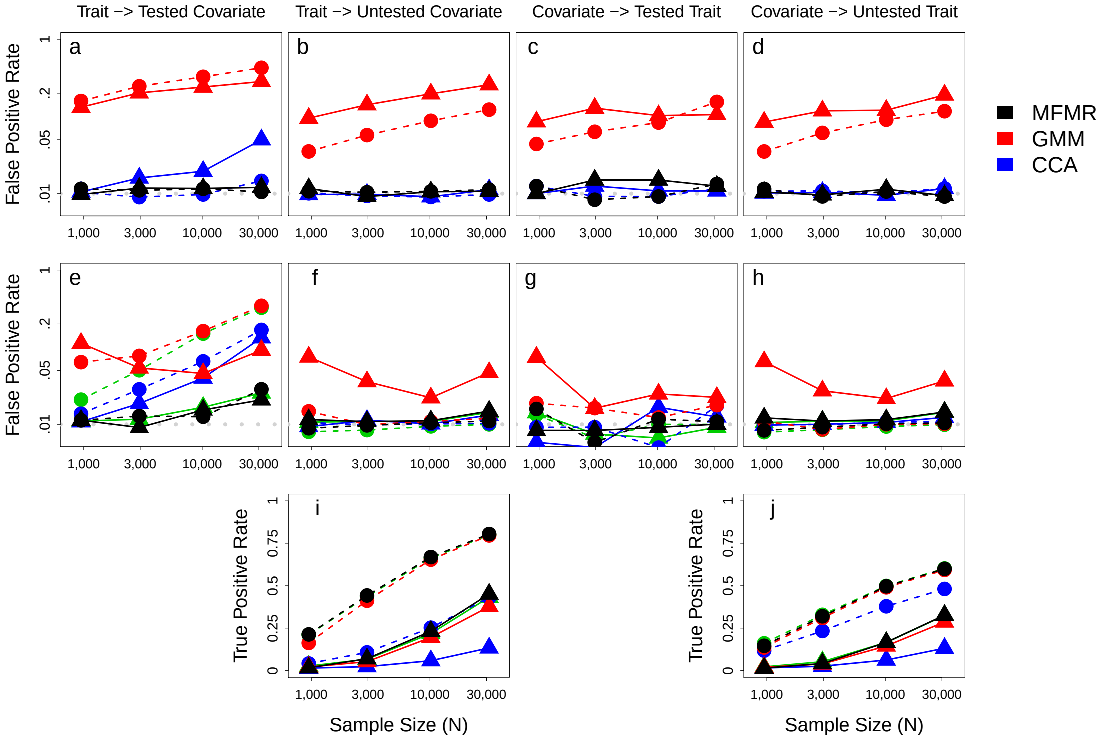

Figure 9: **Simulations where some covariates and traits are swapped.** Simulation modification where decompositions falsely treat a trait as a SNP/covariate (a,b,e,f,i) or vice versa (c,d,g,h,j). (a-d) No genetic or main subtype heterogeneity is simulated, so that the positive heterogeneity associations are unambiguously false. We test both the variable that we misplace (a,c) and the correctly place SNP/covariates and traits (b,d). (e-j) Simulations are drawn as in main text Figure 1, with $K = 2$. (e-h) Tests are shown for the misplaced trait/covariate in (e,g); for the truly homogeneous SNPs in (f,h); and for the truly heterogeneous SNPs in (i,j).

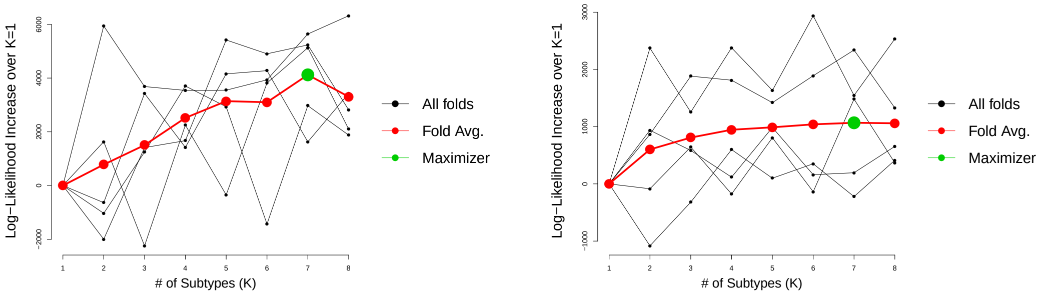

Figure 10: **Out-of-sample likelihood varying K in CONVERGE (left) and METSIM (right).** Samples are split into 5 folds; parameters are fit holding one fold out; the parameters's likelihood is evaluated on the held out fold; and the process is repeated for each fold. The log-likelihoods are shown relative to the baseline likelihood of each fold at $K = 1$; this is analogous to using likelihood ratio statistics to compare a general K to the null with $K = 1$. The average across folds are shown in red, and the maximizer of K is highlighted in green.

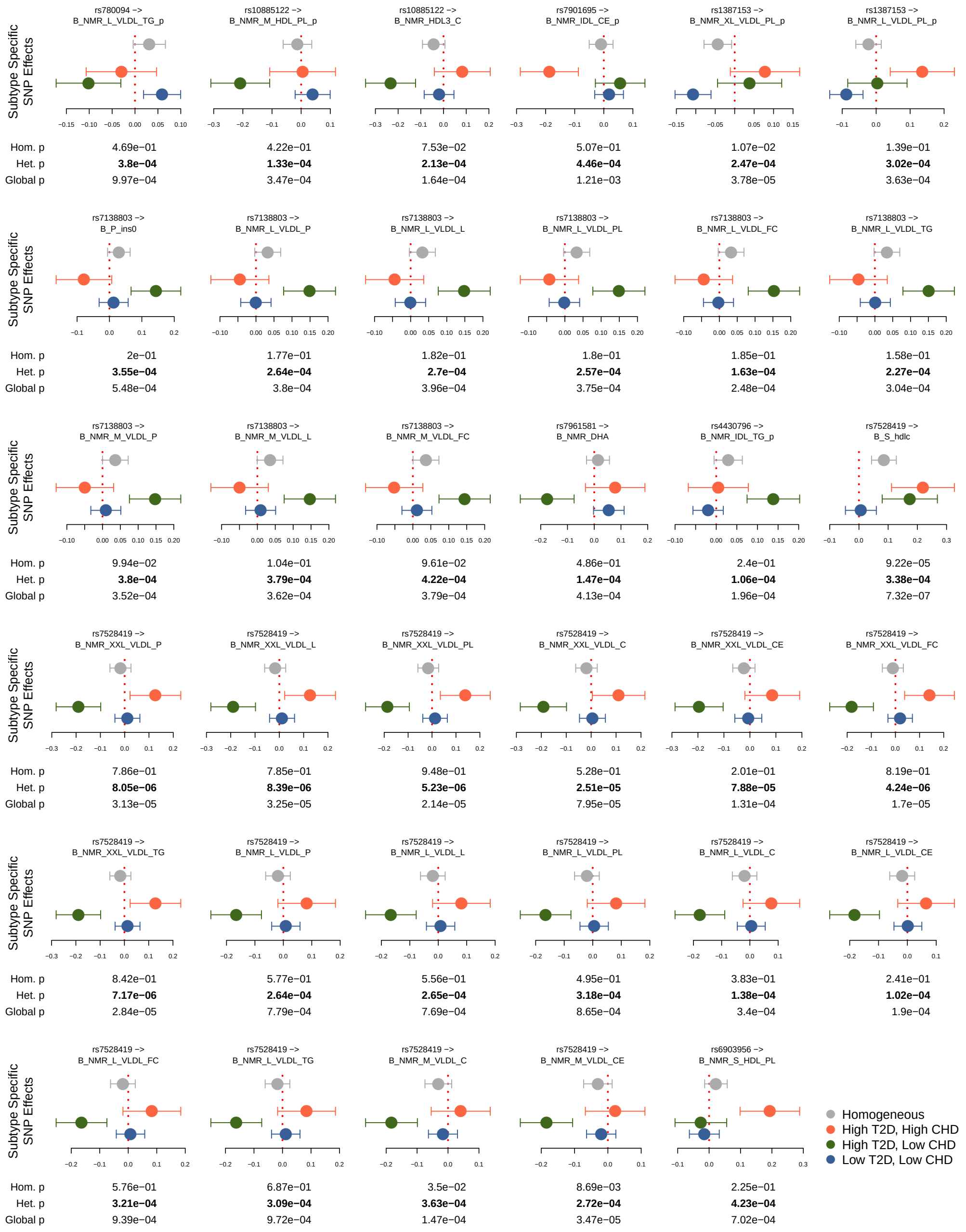

Figure 11: **Metabolic subtype-specific SNP effects across all 228 NMR traits.** SNP-phenotype pairs where the test for effect heterogeneity across subtypes is significant at $p = .05/81$. We test all 228 NMR-based metabolomic traits here rather than using their top PCs as in main Figure 4 and the MFMR decomposition used to learn subtypes. Per-subtype estimates and standard errors are provided in colors as in main Figure 4.

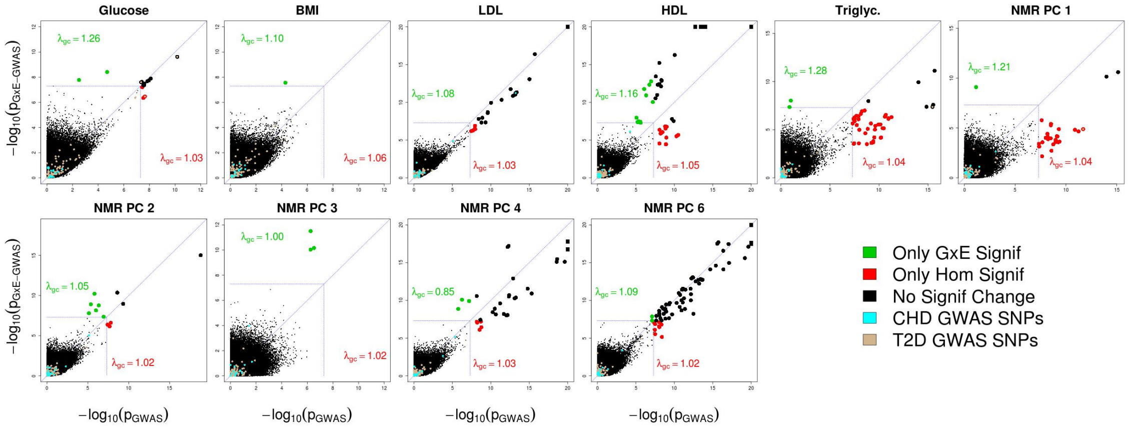

Figure 12: **Comparison of the $-\log_{10}(p)$-values for ordinary GWAS (x-axis) and our novel GxE GWAS (y-axis).** Guide lines are drawn at $p = 5 \times 10^{-8}$, the conventional GWAS threshold. Each point is a SNP, and colors indicate which analyses were significant for the SNP. T2D, CHD, WHR and insulin are omitted because they have no genome-wide significant hits in either analysis; preT2D is omitted because the only hit is shared between both analyses. NMR PC 5 is omitted because it is badly inflated in GxE GWAS ($\lambda_{GC} = 1.63$); this trait has one hit in GWAS.

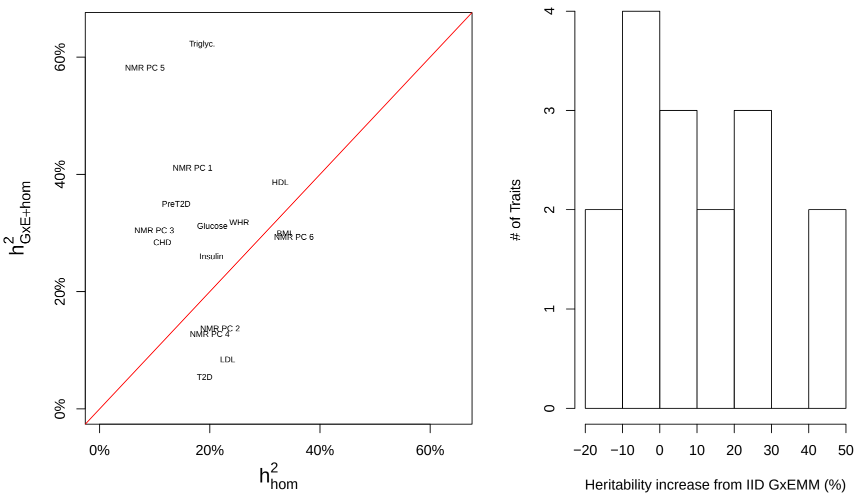

Figure 13: **Comparison of GREML and IID metabolic heritability estimates.** Left: total IID GxEMM heritability (which adds the homogeneous and heterogeneous estimates) compared to the ordinary heritability estimated with GREML. Right: histogram of per-trait heritability increases from replacing GREML with IID GxEMM.

	GWAS	RGWAS		Previous Subtyping					
Clustering	N/A	MFMR		GMM					
Adjusts PCs?	Yes	Yes		No					
Test type	Hom.	Het.	Global	Het.	Global	Het.	Global	Het.	Global
K	1	3	3	2	2	3	3	4	4
Degrees of freedom	1	2	3	1	2	2	3	3	4
T2D	1.03	1.05	1.05	1.82	1.54	44.27	40.47	136.42	131.38
preT2D	1.03	1.07	1.08	0.63	0.72	57.36	53.08	95.30	90.81
CHD	1.04	1.10	1.12	21.71	17.94	12.42	10.66	16.35	14.69
Glucose	1.03	1.29	1.26	0.66	0.75	89.38	84.29	144.17	139.14
Insulin	1.04	1.15	1.15	0.94	0.96	142.72	136.71	Inf	Inf
BMI	1.06	1.06	1.10	0.69	0.79	118.93	113.32	Inf	Inf
LDL	1.03	1.07	1.08	0.95	0.95	56.58	52.32	Inf	Inf
HDL	1.05	1.16	1.16	11.61	9.08	Inf	Inf	Inf	Inf
Trigl.	1.04	1.33	1.28	2.53	2.04	Inf	Inf	Inf	Inf
WHR	1.04	1.09	1.11	0.88	0.92	47.25	43.39	Inf	Inf
NMR PC 1	1.04	1.23	1.21	43.78	38.57	Inf	Inf	Inf	Inf
NMR PC 2	1.02	1.04	1.05	3.55	2.69	106.09	100.63	Inf	Inf
NMR PC 3	1.02	0.98	1.00	Inf	Inf	Inf	Inf	Inf	Inf
NMR PC 4	1.03	0.82	0.85	Inf	Inf	Inf	Inf	Inf	Inf
NMR PC 5	1.00	1.83	1.64	Inf	Inf	Inf	Inf	Inf	Inf
NMR PC 6	1.02	1.07	1.09	Inf	Inf	Inf	Inf	Inf	Inf

Table 1: λ_{GC} **for GWAS, MFMR GxE GWAS, and GMM GxE GWAS.** GWAS means the standard regression approach conditioning on known covariates and genetic PCs. RGWAS is our approach, which uses covariate-aware clusters (MFMR) and tests for genetic variant effect heterogeneity (Het) or globally for any genetic effect (Global). "Previous Subtyping" is like RGWAS, except using covariate-unaware clustering (GMM) and heterogeneity tests. "Inf" means our calculations suffered numerical problems, meaning that λ_{GC} is very large. We do not perform SNP tests on NMR PC 5.

	MFMR `droptest`			+Condition on T2D		
	K=2	K=3	K=4	K=2	K=3	K=4
T2D	0.197	0.495	0.770	1.000	1.000	1.000
preT2D	0.230	0.125	0.408	0.414	0.075	NA
CHD	0.871	0.340	0.282	0.844	0.427	0.805
BMI	0.976	0.000	0.000	0.393	0.000	0.000
Glucose	0.218	0.046	0.858	0.416	0.018	0.709
Insulin	0.291	0.042	0.770	0.475	0.029	0.791
LDL	0.318	0.038	0.005	0.281	0.079	0.002
HDL	0.139	0.075	0.020	0.231	0.045	0.008
Trigl.	0.086	0.345	0.079	0.125	0.138	0.094
WHR	0.233	0.251	0.748	0.357	0.178	0.667
NMR PC 1	0.053	0.523	0.044	0.067	0.220	0.061
NMR PC 2	0.008	0.006	0.056	0.016	0.003	0.089
NMR PC 3	0.000	0.014	0.006	0.000	0.012	0.003
NMR PC 4	0.787	0.651	0.316	0.863	0.728	0.455
NMR PC 5	0.520	0.437	0.092	0.511	0.425	0.088
NMR PC 6	0.011	0.218	0.014	0.011	0.302	0.012

Table 2: **Statin effect heterogeneity test for $K \in \{2, 3, 4\}$ and the 16 traits used to define clusters with MFMR.** The "MFMR `droptest`" columns test using the large-effect covariate test implemented in the `rgwas` R package and described in the Methods in the main text. The "+Condition on T2D" columns run the same linear model as in `droptest`, except that T2D status is additionally included as a covariate; this analysis is performed to add confidence that the heterogeneous statin effect on glucose is not merely driven by simple confounding from T2D status.

References

[1] Xiao-Li Meng and Donald B Rubin. "Maximum likelihood estimation via the ECM algorithm: A general framework". *Biometrika* 80.2 (1993), pp. 267–278.

[2] Jerome Friedman, Trevor Hastie, and Rob Tibshirani. "Regularization Paths for Generalized Linear Models via Coordinate Descent." *Journal of Statistical Software* 33.1 (2010), pp. 1–22.

[3] Rahul Mazumder and Trevor Hastie. "The graphical lasso: New insights and alternatives". *Electronic Journal of Statistics* 6.0 (2012), pp. 2125–2149.

[4] David Golan, Eric S Lander, and Saharon Rosset. "Measuring missing heritability: inferring the contribution of common variants." *Proceedings of the National Academy of Sciences of the United States of America* 111.49 (2014), E5272–81.

[5] Bruce Lindsay and Jiawei Liu. "Model Assessment Tools for a Model False World". *Statistical Science* 24.3 (2009), pp. 303–318.

[6] Andrew Dahl, Vincent Guillemot, Joel Mefford, Hugues Aschard, and Noah Zaitlen. "Adjusting For Principal Components Of Molecular Phenotypes Induces Replicating False Positives". *BioRxiv* (2017), p. 120899.
