# Supplementary figures and images for: Reverse GWAS: Using genetics to identify and model phenotypic subtypes
Source: PLoS Genet. 2019 Apr 5;15(4):e1008009. doi: 10.1371/journal.pgen.1008009 (PMC6469799; doi:10.1371/journal.pgen.1008009)

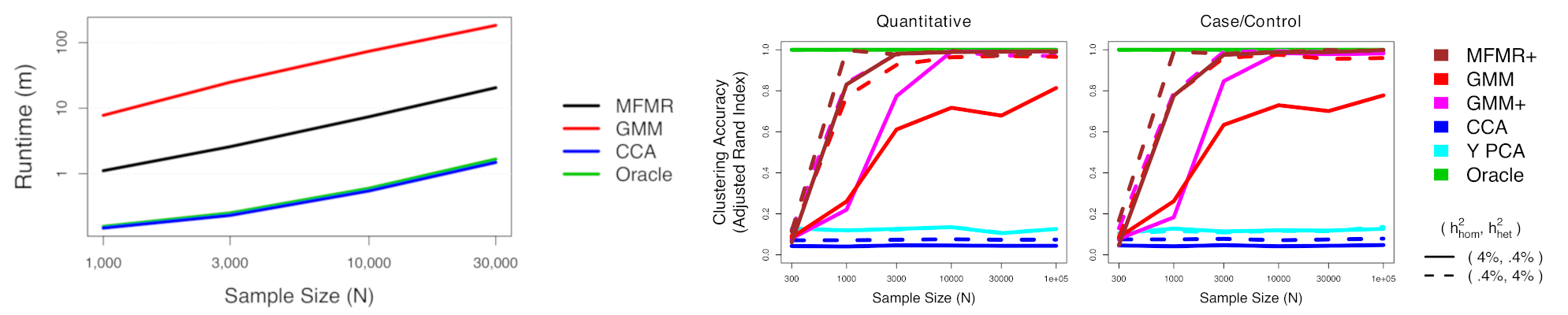

Supplement: S1 Fig — Left: Average running times in main Fig 1 (excluding failed GMM runs). Right: Clustering accuracy for simulations without (‘Quantitative’, as in main Fig 1) and with ascertainment (‘Case/Control’, as in S2 Fig). We measure accuracy with adjusted Rand index, which varies from 0 (random guessing) to 1 (exact match). We compute the index only across pairs from a random 300 subsamples, reducing computation roughly ≈ 105-fold when N = 100, 000. Accuracies are estimated for roughly 300 simulations per point in the plot. MFMR+ is shown for simplicity because MFMR gives different clusters per tested SNP. (TIF) [file pgen.1008009.s002.tif]

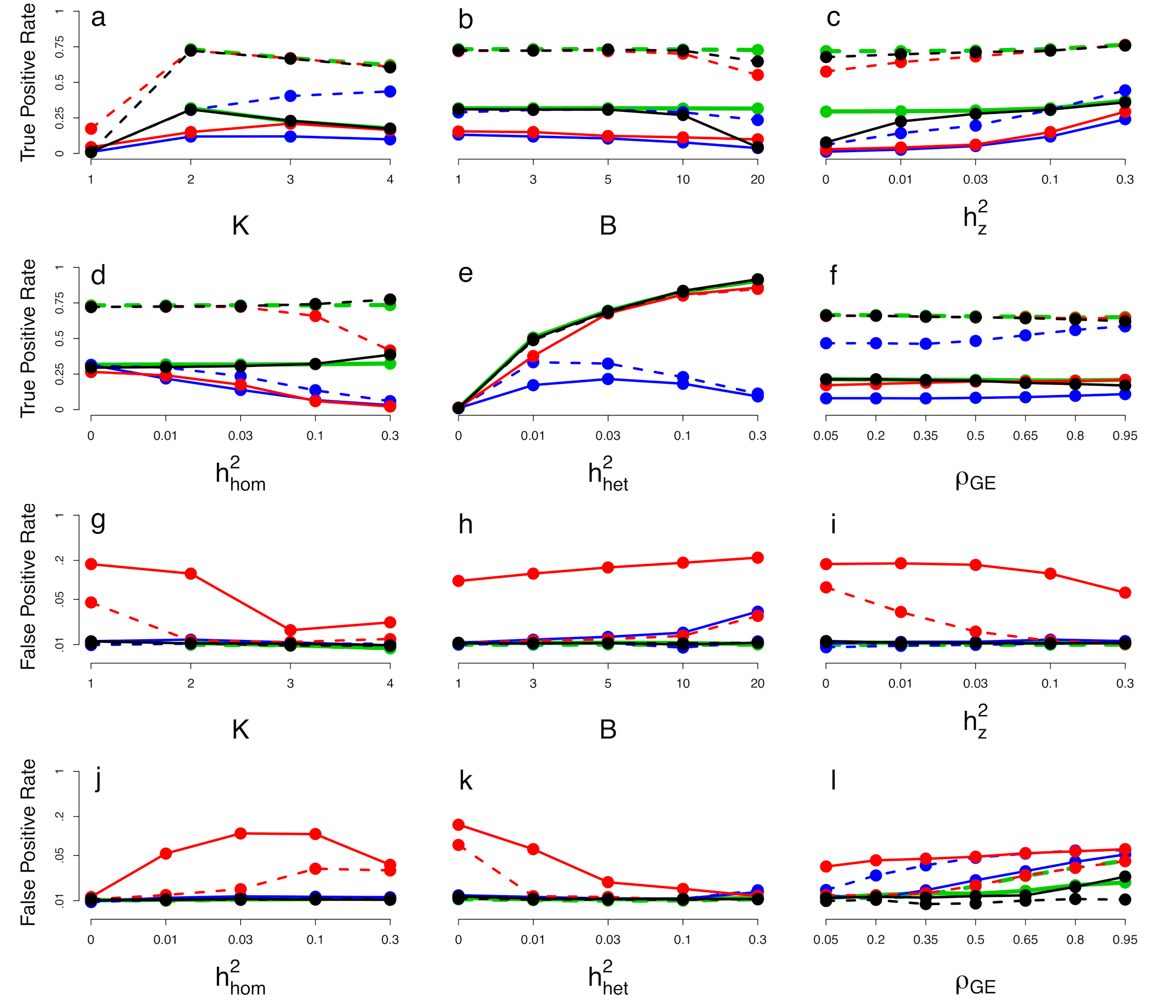

Supplement: S2 Fig — Tests for truly heterogeneous SNPs are shown in the top 6 panels (a-f), and the corresponding tests for SNPs with only homogeneoues effects are shown in the below 6 panels (g-l). K is the number of true, simulated subtypes and B is the number of binary traits. ρGE is the gene-subtype correlation term, with ρGE = 0 giving non-heritable subtype statuses and ρGE = 1 giving perfectly heritable subtypes. hhom2, hhet2, and hz2 are the variances explained by homogeneous SNPs, heterogeneous SNPs, and main subtype effects, respectively. As in main Fig 1, solid lines have (hhom2,hhet2)=(4%,.4%), and dashed lines are reversed; in (d,e), line types define only the h2 term not governed by the x-axis. In (a), all methods fit K = 2 subtypes; there is no true heterogeneity for K = 1, where the oracle is not defined, and for K > 1 and the oracle picks a true cluster at random. Generally, increasing the heterogeneous factors (hhet2 and hz2) makes subtyping easier, while increasing hhom2 makes subtyping harder. (TIF) [file pgen.1008009.s003.tif]

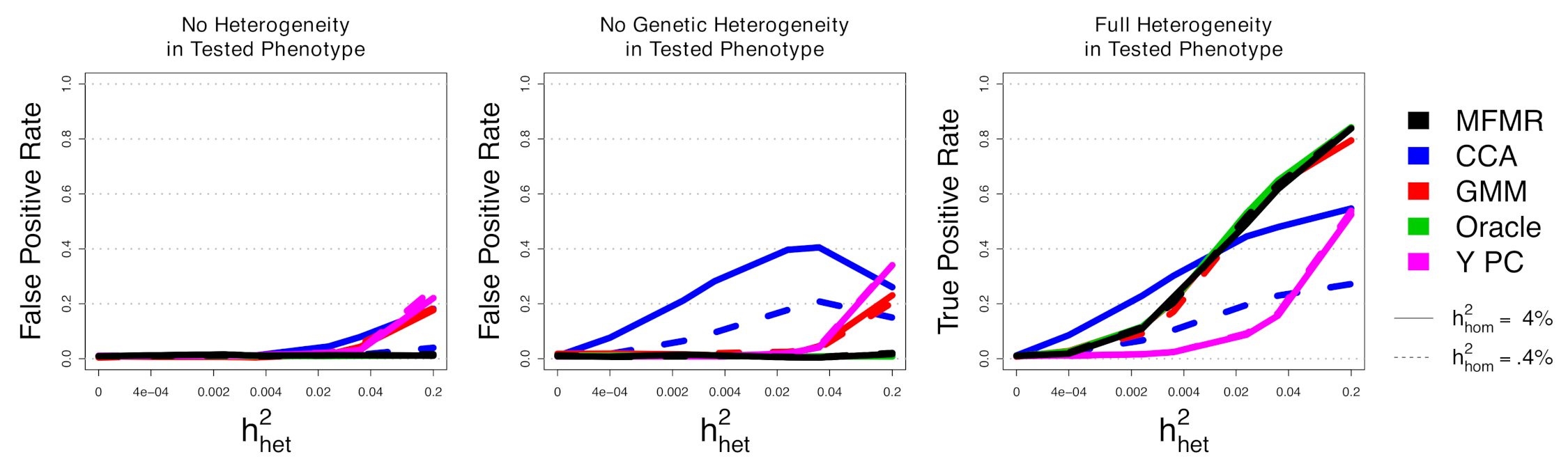

Supplement: S3 Fig — Left: the tested trait has no genetic heterogeneity or main subtype effect. Center: the tested trait has only a main subtype effect but no heterogeneity. Right: the full heterogeneity simulation. Linear subtype estimators (CCA and Y PC) are not trait-specific. (TIF) [file pgen.1008009.s004.tif]

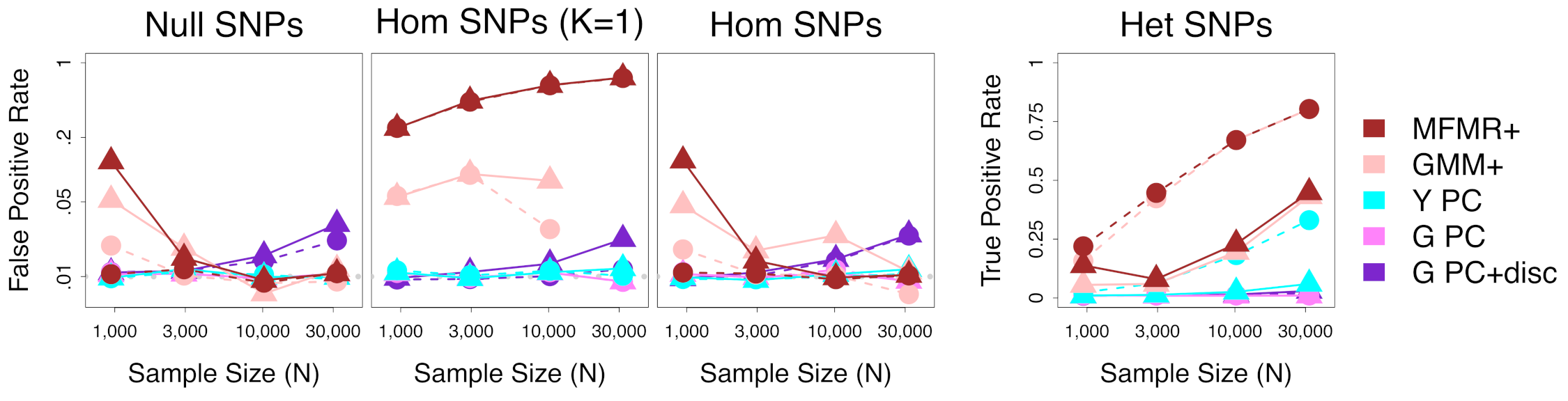

Supplement: S4 Fig — MFMR+ varies MFMR by treating the tested SNP as heterogeneous. GMM+ varies GMM by including the SNPs as traits when clustering. As expected, MFMR+ and GMM+ are miscalibrated. GMM+ often fails to converge, especially for N ≥ 10, 000 (we evaluate only the converged runs). The other methods, with low power, define subtypes as the top PC of Y or G, optionally thresholded to be binary (“G PC+disc”). (TIF) [file pgen.1008009.s005.tif]

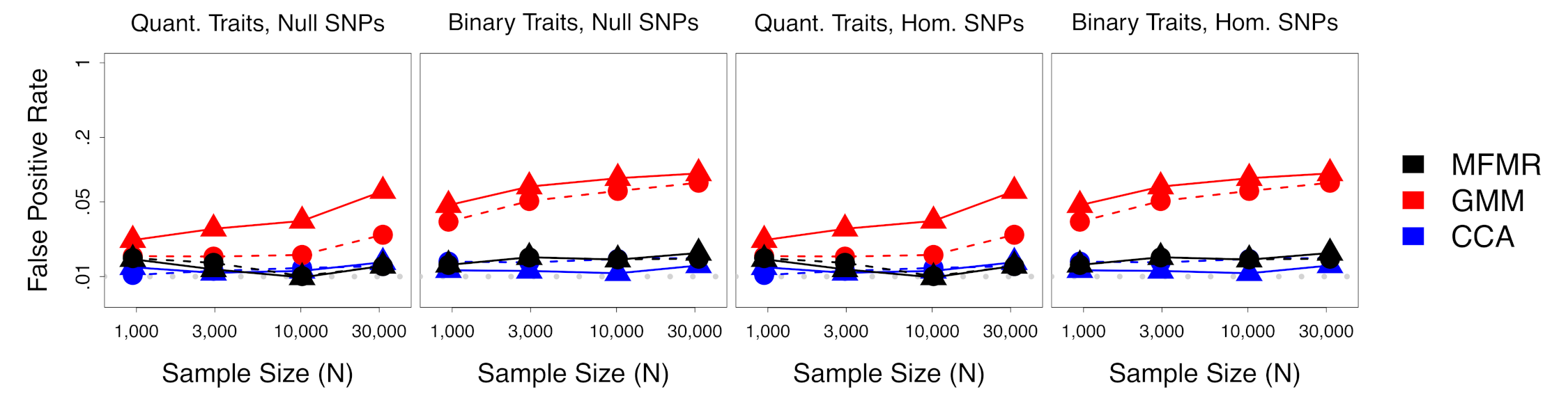

Supplement: S5 Fig — Purely homogeneoues simulations, without subtypes, where the noise, ϵ, has marginal t5 distributions. ϵ is simulated by drawing i.i.d. t5-distributed random variables, arranging into an N × P matrix, and then right-multiplying with Σ1/2, where Σ is the noise covariance matrix and is drawn as in the main simulations in main Fig 1. (TIF) [file pgen.1008009.s006.tif]

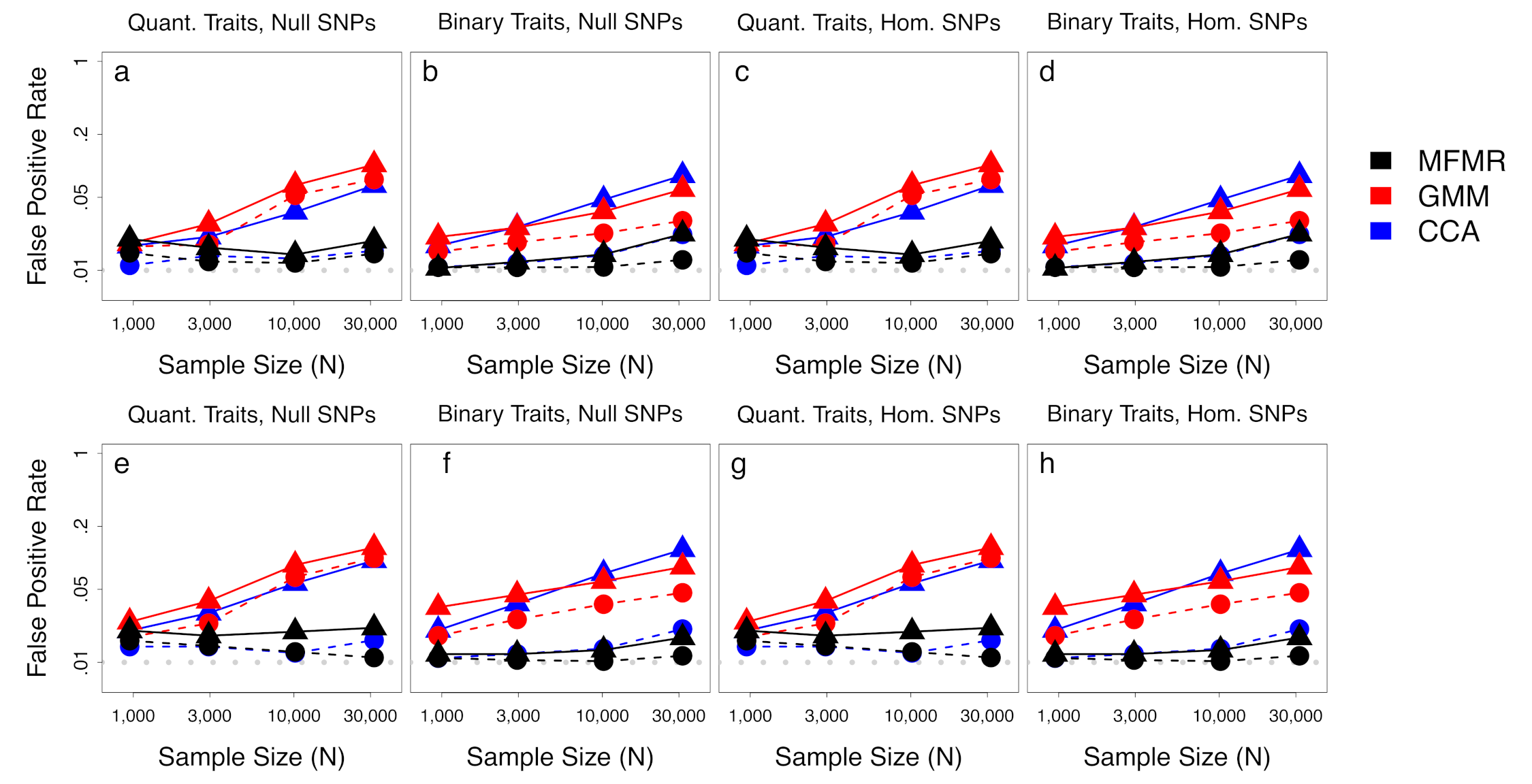

Supplement: S6 Fig — Purely homogeneoues simulations, without subtypes, where SNPs truly have a non-linear effect. In (a-d), the SNPs are squared before use in MFMR, so that the true SNP and the utilized covariate (i.e. SNP2) have zero correlation. In (e-h), the true SNPs are exponentiated before inclusion in MFMR, so the true SNP effect is log-linear. Results are partitioned by whether the tested traits are quantitative or binary, as well as by whether the true SNP effect is null or homogeneous. (TIF) [file pgen.1008009.s007.tif]

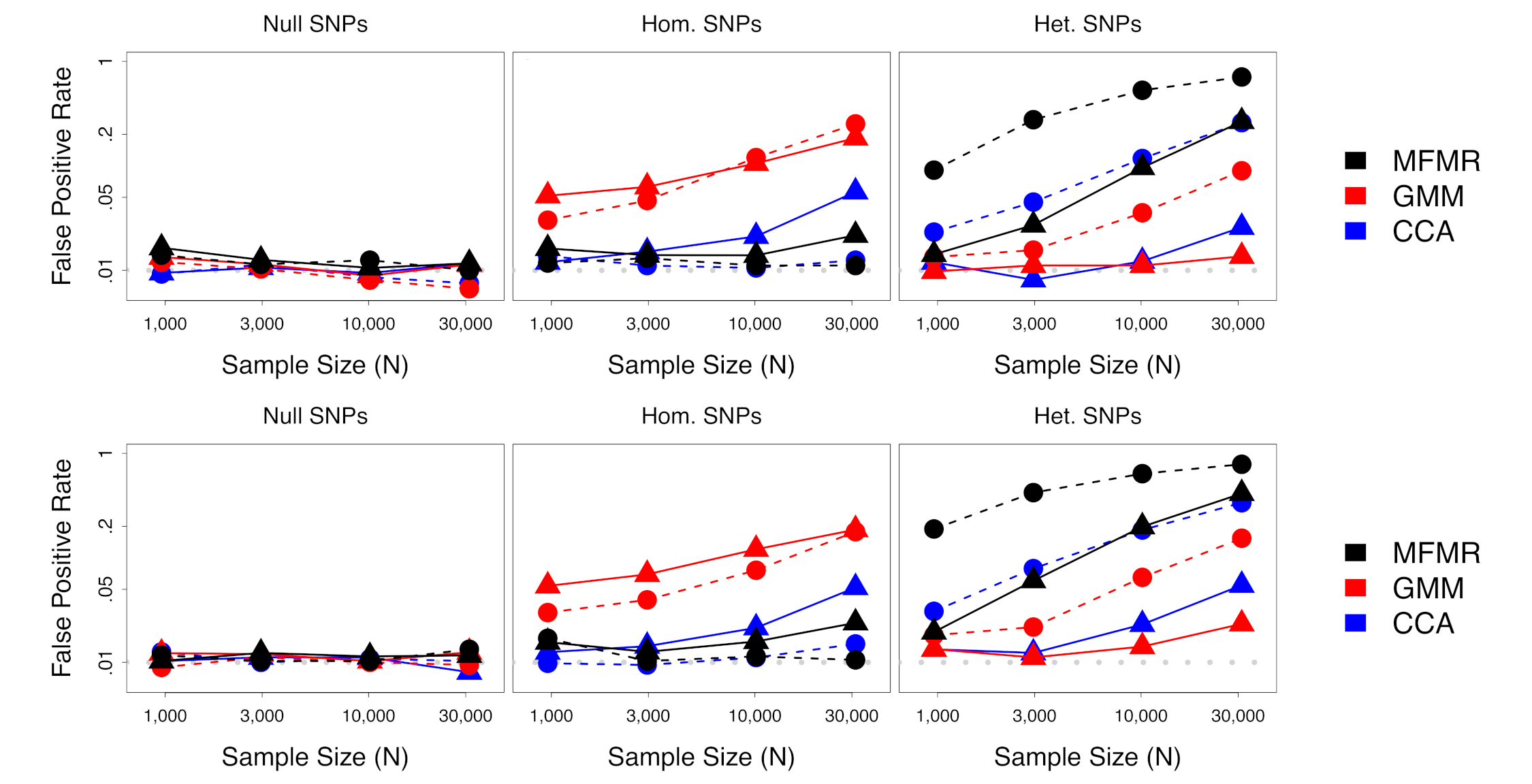

Supplement: S7 Fig — z is chosen to be Gaussian. Top: Effect sizes are chosen so that power roughly matches main Fig 1; it is not trivial to directly convert effect sizes from the discrete z simulations. Bottom: All heterogeneoues effect sizes are doubled relative to top panels. (TIF) [file pgen.1008009.s008.tif]

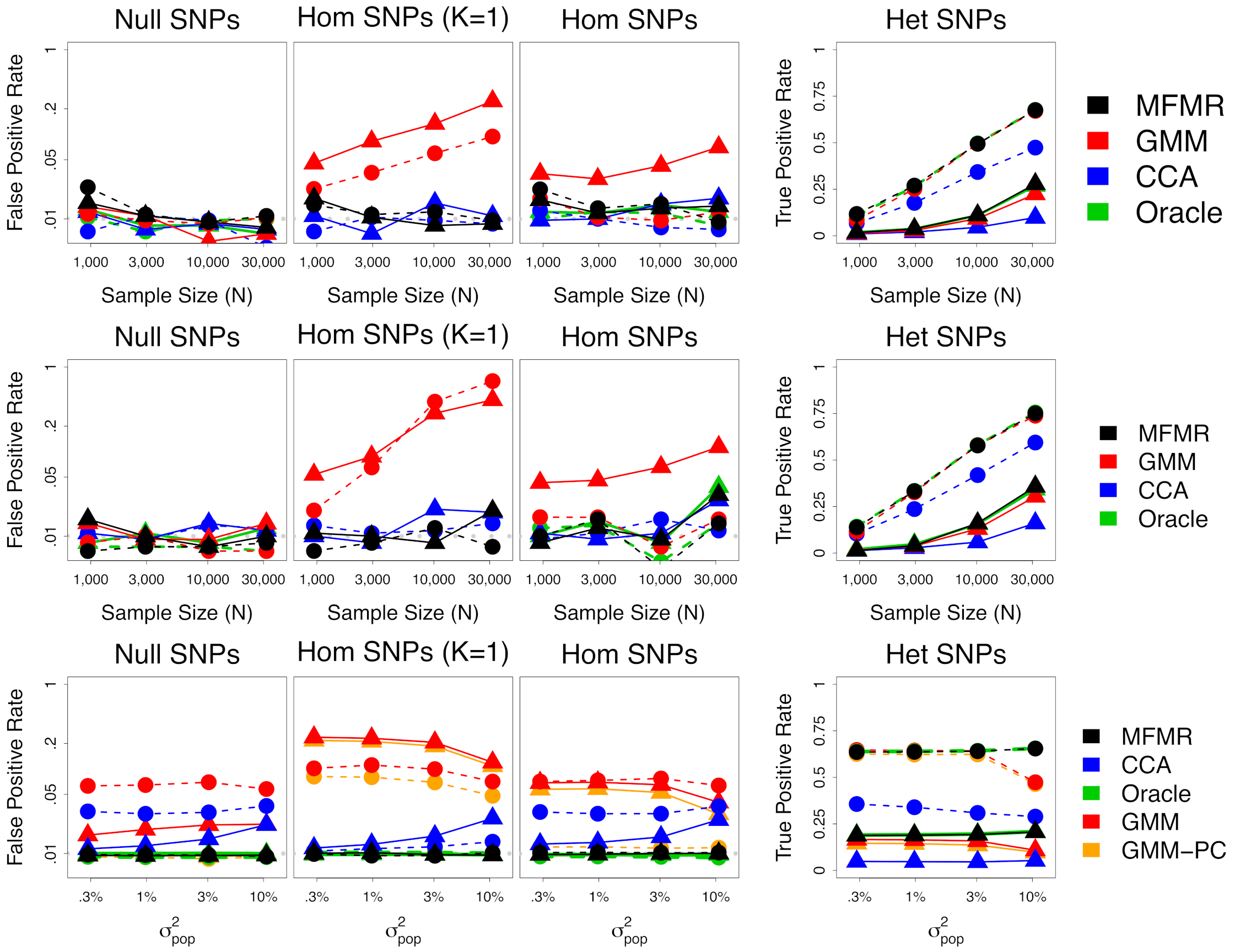

Supplement: S8 Fig — Top: SNP effect heterogeneity tests are applied to binary traits, not quantitative traits as in main Fig 1. Even though GMM only clusters the quantitative traits, tests for the (correlated) binary traits are miscalibrated. Middle: a 20% population prevalence binary trait is ascertained to have 50% in-sample prevalence and then tested. Bottom: population structure is added and MFMR, Oracle and GMM-PC test conditional on three genetic PCs; GMM and GMM-PC use the same subtype estimator. (TIF) [file pgen.1008009.s009.tif]

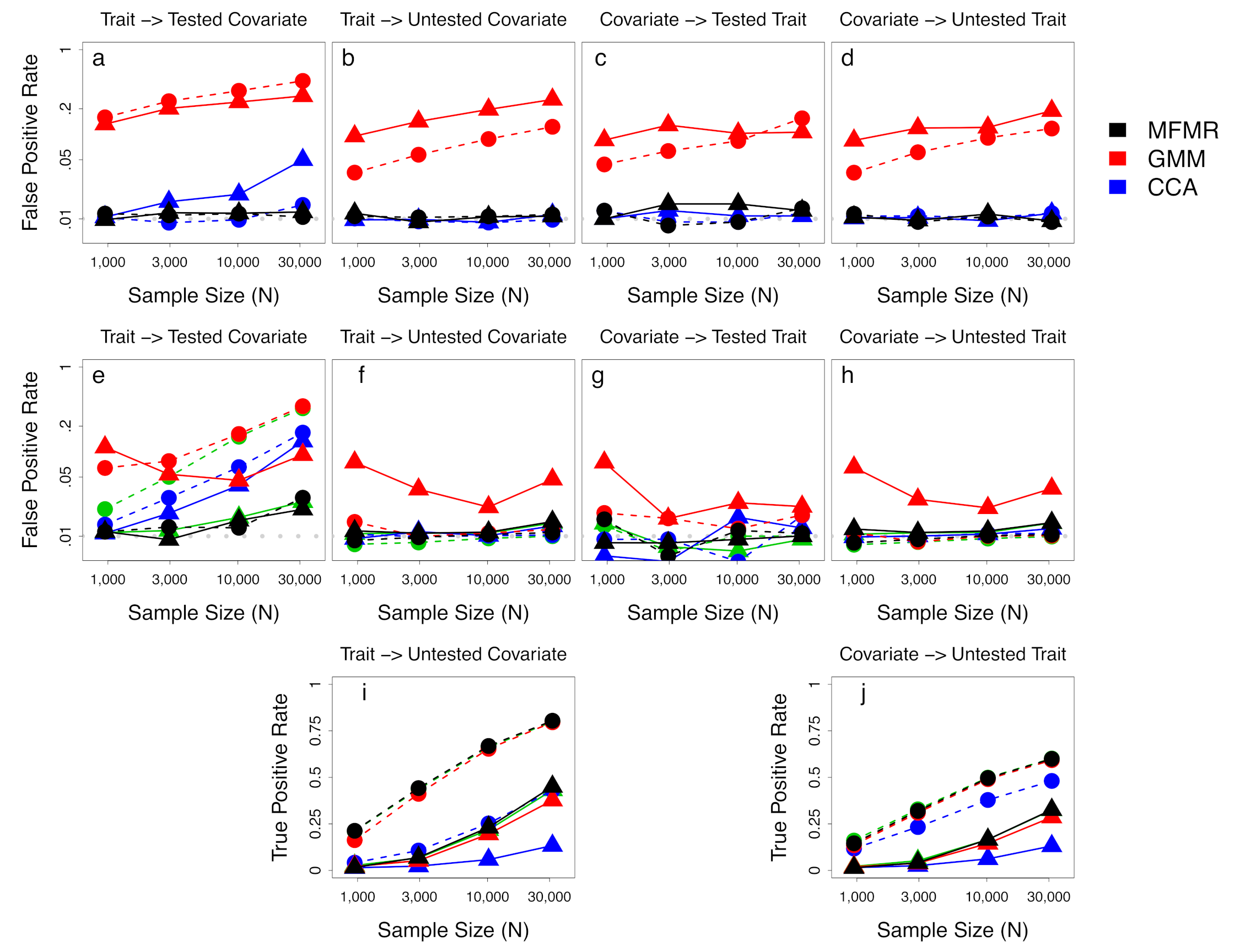

Supplement: S9 Fig — Simulation modification where decompositions falsely treat a trait as a SNP/covariate (a,b,e,f,i) or vice versa (c,d,g,h,j). (a-d) No genetic or main subtype heterogeneity is simulated, so that the positive heterogeneity associations are unambiguously false. We test both the variable that we misplace (a,c) and the correctly place SNP/covariates and traits (b,d). (e-j) Simulations are drawn as in main text Fig 1, with K = 2. (e-h) Tests are shown for the misplaced trait/covariate in (e,g); for the truly homogeneous SNPs in (f,h); and for the truly heterogeneous SNPs in (i,j). (TIF) [file pgen.1008009.s010.tif]

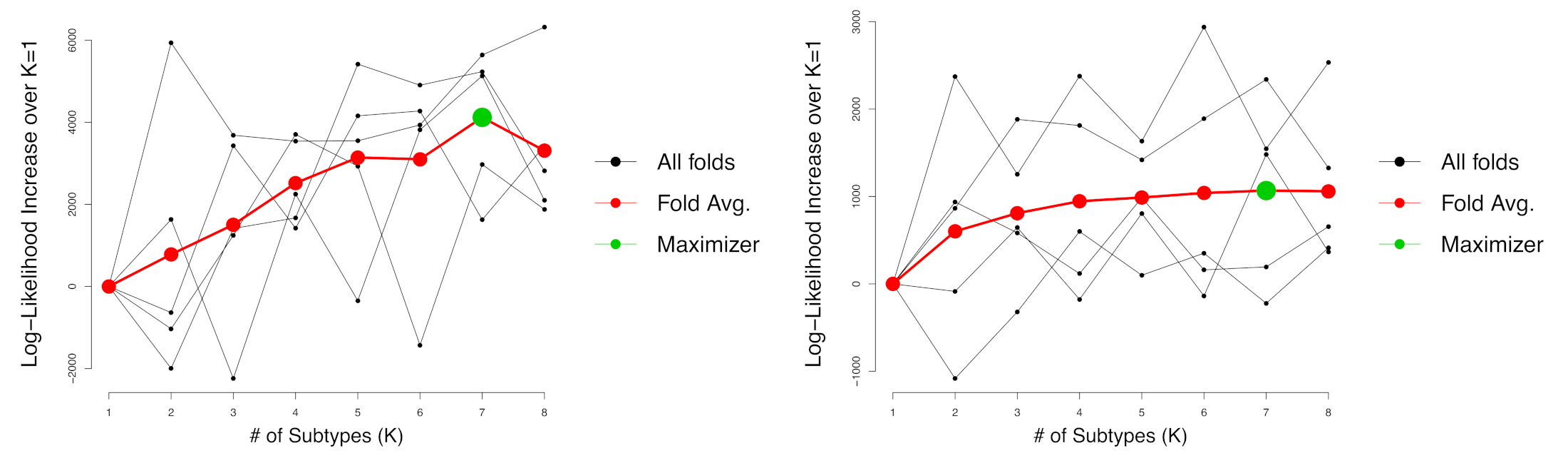

Supplement: S10 Fig — Samples are split into 5 folds; parameters are fit holding one fold out; the parameters’s likelihood is evaluated on the held out fold; and the process is repeated for each fold. The log-likelihoods are shown relative to the baseline likelihood of each fold at K = 1; this is analogous to using likelihood ratio statistics to compare a general K to the null with K = 1. The average across folds are shown in red, and the maximizer of K is highlighted in green. (TIF) [file pgen.1008009.s011.tif]

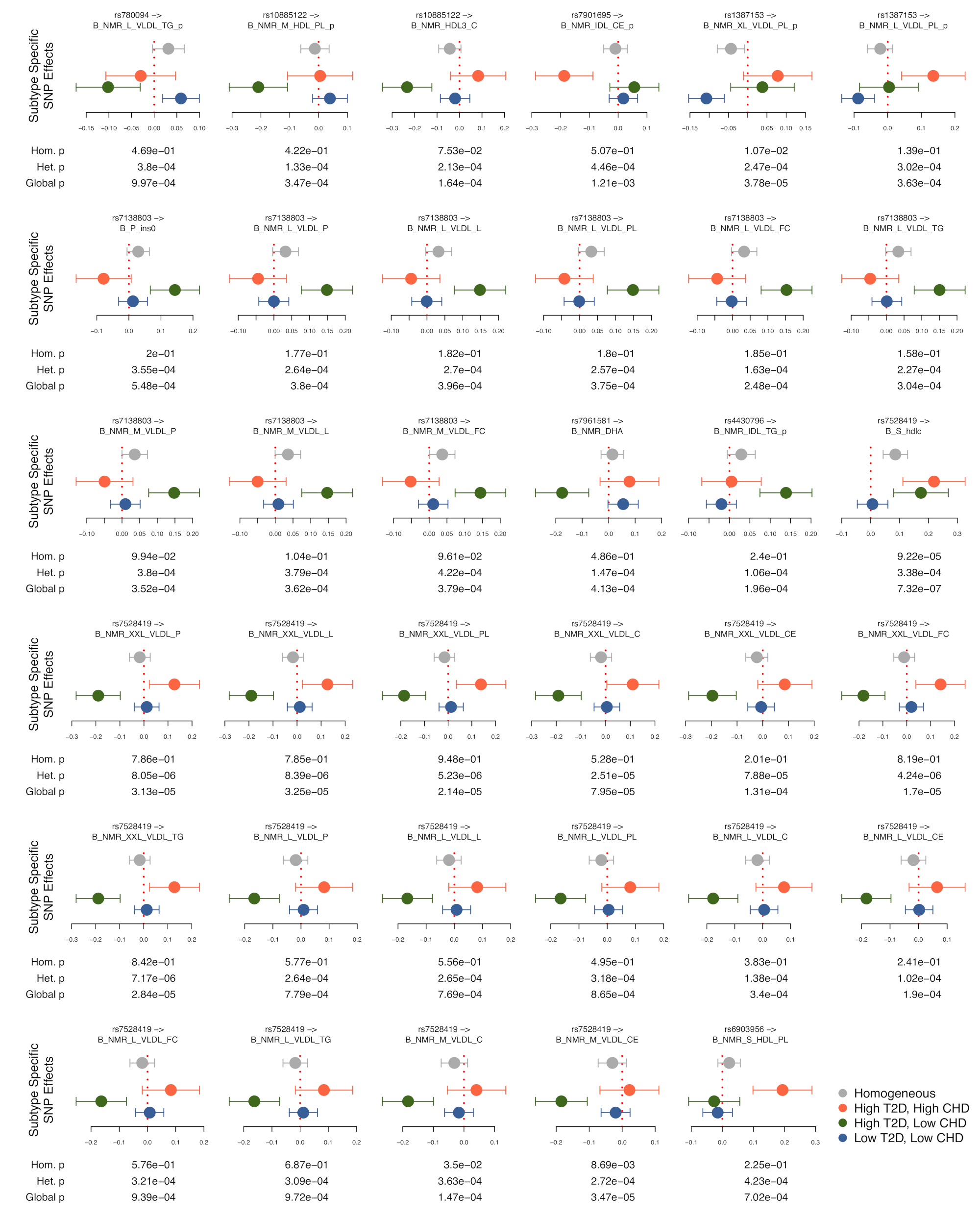

Supplement: S11 Fig — SNP-phenotype pairs where the test for effect heterogeneity across subtypes is significant at p = .05/81. We test all 228 NMR-based metabolomic traits here rather than using their top PCs as in main Fig 4 and the MFMR decomposition used to learn subtypes. Per-subtype estimates and standard errors are provided in colors as in main Fig 4. (TIF) [file pgen.1008009.s012.tif]

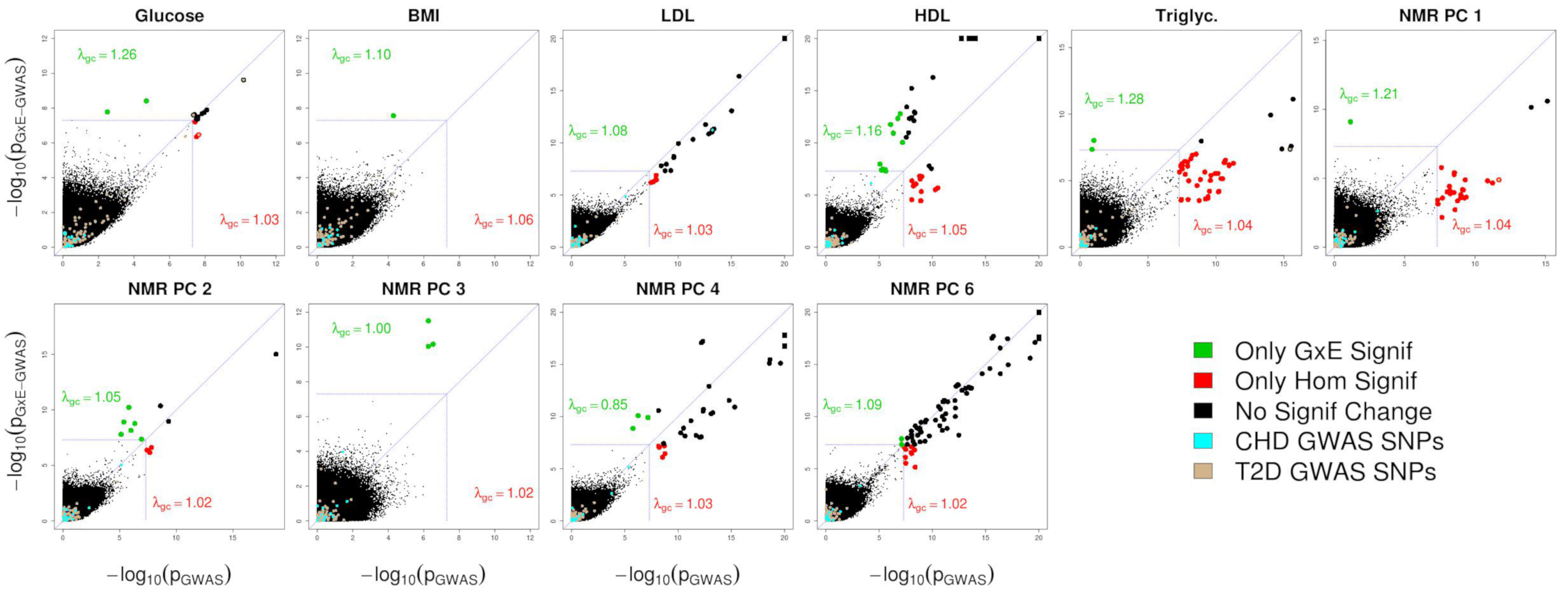

Supplement: S12 Fig — Guide lines are drawn at p = 5 × 10^−8^, the conventional GWAS threshold. Each point is a SNP, and colors indicate which analyses were significant for the SNP. T2D, CHD, WHR and insulin are omitted because they have no genome-wide significant hits in either analysis; preT2D is omitted because the only hit is shared between both analyses. NMR PC 5 is omitted because it is badly inflated in GxE GWAS (λGC = 1.83); this trait has one hit in GWAS. (TIF) [file pgen.1008009.s013.tif]

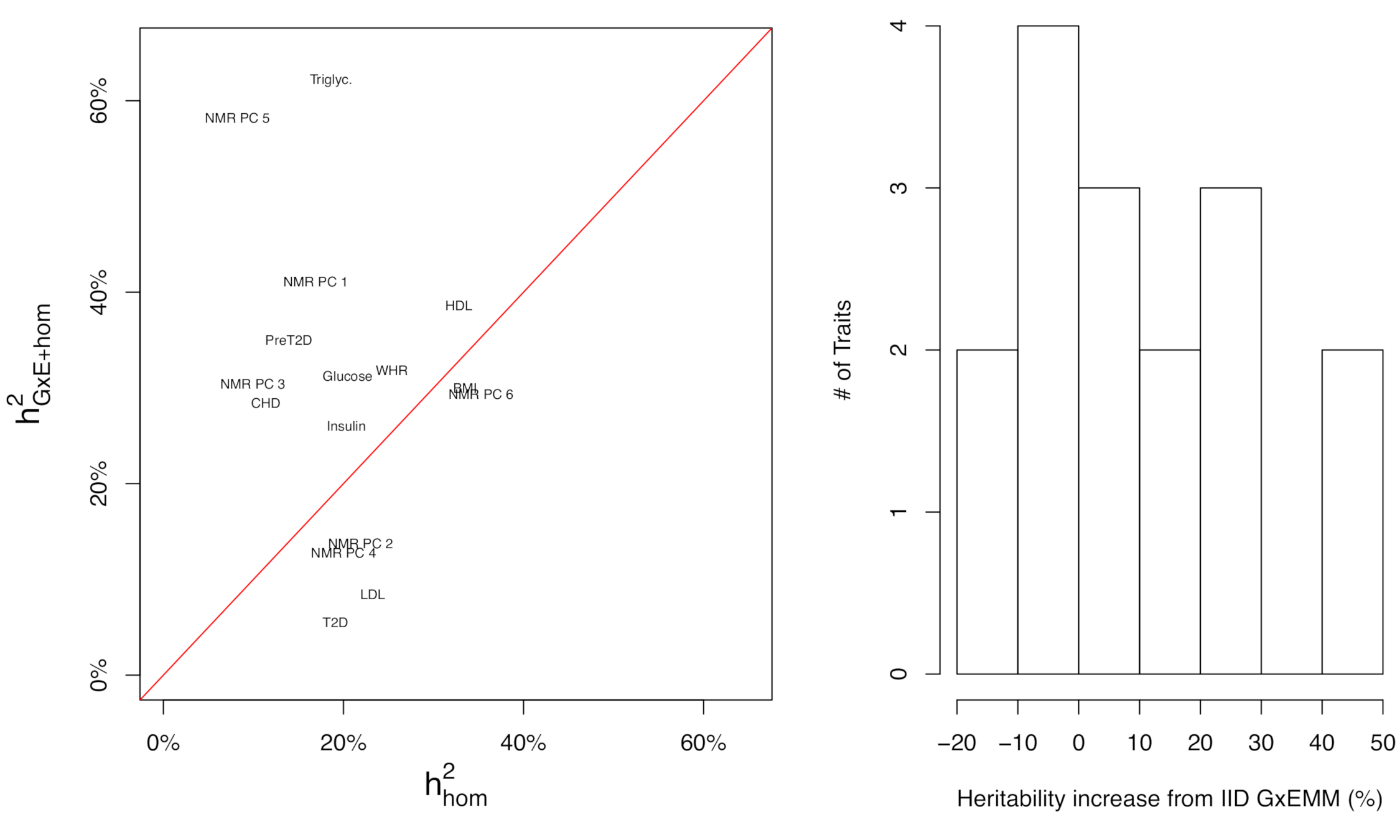

Supplement: S13 Fig — Left: total IID GxEMM heritability (which adds the homogeneous and heterogeneous estimates) compared to the ordinary heritability estimated with GREML. Right: histogram of per-trait heritability increases from replacing GREML with IID GxEMM. (TIF) [file pgen.1008009.s014.tif]
